# Streamlined human antibody generation and optimization by exploiting designed immunoglobulin loci in a B cell line

**DOI:** 10.1038/s41423-020-0440-9

**Published:** 2020-05-26

**Authors:** Hidetaka Seo, Hitomi Masuda, Kenjiro Asagoshi, Tomoaki Uchiki, Shigehisa Kawata, Goh Sasaki, Takashi Yabuki, Shunsuke Miyai, Naoki Takahashi, Shu-ichi Hashimoto, Atsushi Sawada, Aki Takaiwa, Chika Koyama, Kanako Tamai, Kohei Kurosawa, Ke-Yi Lin, Kunihiro Ohta, Yukoh Nakazaki

**Affiliations:** 1 Department of Life Sciences, Graduate School of Arts and Sciences, The University of Tokyo, Tokyo, Japan; 2 Research Laboratories, Chiome Bioscience Inc, Tokyo, Japan

**Keywords:** Immunoglobulin rearrangements, homologous recombination, antibody therapeutics, antibody engineering, drug discovery, Drug discovery, Cell biology, Immunology

## Abstract

Monoclonal antibodies (mAbs) are widely utilized as therapeutic drugs for various diseases, such as cancer, autoimmune diseases, and infectious diseases. Using the avian-derived B cell line DT40, we previously developed an antibody display technology, namely, the ADLib system, which rapidly generates antigen-specific mAbs. Here, we report the development of a human version of the ADLib system and showcase the streamlined generation and optimization of functional human mAbs. Tailored libraries were first constructed by replacing endogenous immunoglobulin genes with designed human counterparts. From these libraries, clones producing full-length human IgGs against distinct antigens can be isolated, as exemplified by the selection of antagonistic mAbs. Taking advantage of avian biology, effective affinity maturation was achieved in a straightforward manner by seamless diversification of the parental clones into secondary libraries followed by single-cell sorting, quickly affording mAbs with improved affinities and functionalities. Collectively, we demonstrate that the human ADLib system could serve as an integrative platform with unique diversity for rapid de novo generation and optimization of therapeutic or diagnostic antibody leads. Furthermore, our results suggest that libraries can be constructed by introducing exogenous genes into DT40 cells, indicating that the ADLib system has the potential to be applied for the rapid and effective directed evolution and optimization of proteins in various fields beyond biomedicine.

## Introduction

In the past three decades, multiple approaches have been taken to reduce the immunogenicity of mAbs for therapeutic usage.^1,2^ Antibody humanization, in which the complementarity-determining regions (CDRs) of non-human-derived mAbs are grafted into human scaffolds,^3^ has been widely employed. Alternatively, in vitro display technologies relying on phage, yeast, or mammalian cells to display antibody fragments, such as antigen-binding fragments (Fabs) and single-chain variable fragments (scFvs), or full-length IgGs containing human variable (V) regions are powerful tools to identify lead candidate sequences of human origin.^4–13^ In contrast to conventional animal immunization, hybridomas created from the spleen cells of genetically engineered mice carrying human immunoglobulin loci^1,14–17^ can be applied for de novo generation of human antibodies.^2^ Different transgenic animals, such as chickens harboring human antibody variable (V) region genes, have also been developed.^18,19^ Despite the invention of these technologies to generate low-immunogenicity therapeutic mAbs, bottlenecks still remain to be resolved.^2^ For example, antibody humanization is a laborious process typically performed on a case-by-case basis and often results in unpredictable changes in specificities or affinities for antigens.^20^ Due to the limitation of Fabs or scFvs being expressed, further genetic engineering is required to reformat isolated antibody fragments displayed by antigen-specific phages into complete IgG molecules. For affinity maturation of the antibodies, these conventional methods often need additional rounds of diversification-selection cycles,^5,21^ prolonging the overall discovery timeline. For de novo antibody generation platforms, it usually takes months to establish stable hybridomas, and most importantly, there are antigens that are difficult to raise antibodies against due to immune tolerance. On the other hand, in the case of chickens harboring human V regions, the IgM, IgY, and IgA formats of immunoglobulin heavy chain constant regions are produced after immunization, thereby necessitating the cloning of each V region gene into the expression vector followed by introduction into culture cells to obtain human IgGs, which is time consuming and may alter the activities of mAbs.^18,19^

**Fig. 1:**
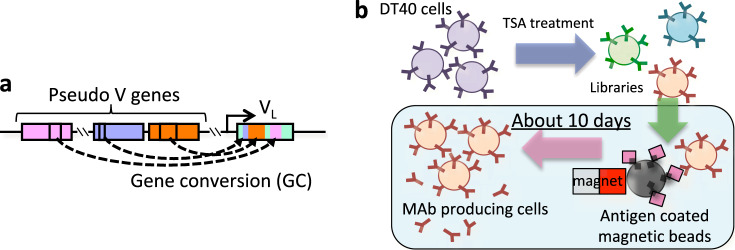
The diversification mechanism of avian immunoglobulin genes and the principle of the ADLib system. **a** Diversification of avian immunoglobulin genes by GC. Partial sequences of the pseudogenes are transferred to the functional V region by homologous recombination. GC is a unidirectional event in which the functional V region is altered while the pseudogenes remain unchanged. **b** Principle of the ADLib system. DT40 cells are cultured in the presence of TSA to generate diversified cell-based mAb libraries. Antigen-specific clones can be isolated by, for example, antigen-conjugated magnetic beads. The isolated cells are expanded, and the antigen-specific mAbs are recovered in culture supernatants. The process, from magnetic bead selection to the recovery of the mAbs, can be completed in ~10 days

We previously reported a rapid method to generate mAbs using an avian-derived B cell line.^22–24^ Avian immunoglobulin loci contain only a single functional variable region for each of the heavy chain (HC) and light chain (LC), whereas clusters of pseudogenes, which show homology to the functional V region, are present in the near-upstream region. To generate diversity, copies of pseudogenes are transferred to the functional V region via unidirectional gene conversion (GC) (a type of homologous recombination) (Fig. 1a).^25^ DT40 is a chicken-derived B cell line in which GC continuously occurs at the immunoglobulin loci.^26^ We discovered that this GC frequency is markedly enhanced when DT40 cells are treated with the histone deacetylase inhibitor trichostatin A (TSA). Using TSA-stimulated GC, an autonomously diversifying library (ADLib) was generated, and clones expressing antigen-specific mAbs can be enriched within ~10 days^27–30^ (Fig. 1b). However, as the original ADLib system expresses chicken IgMs, humanization is still required for subsequent development.^29^

**Fig. 2:**
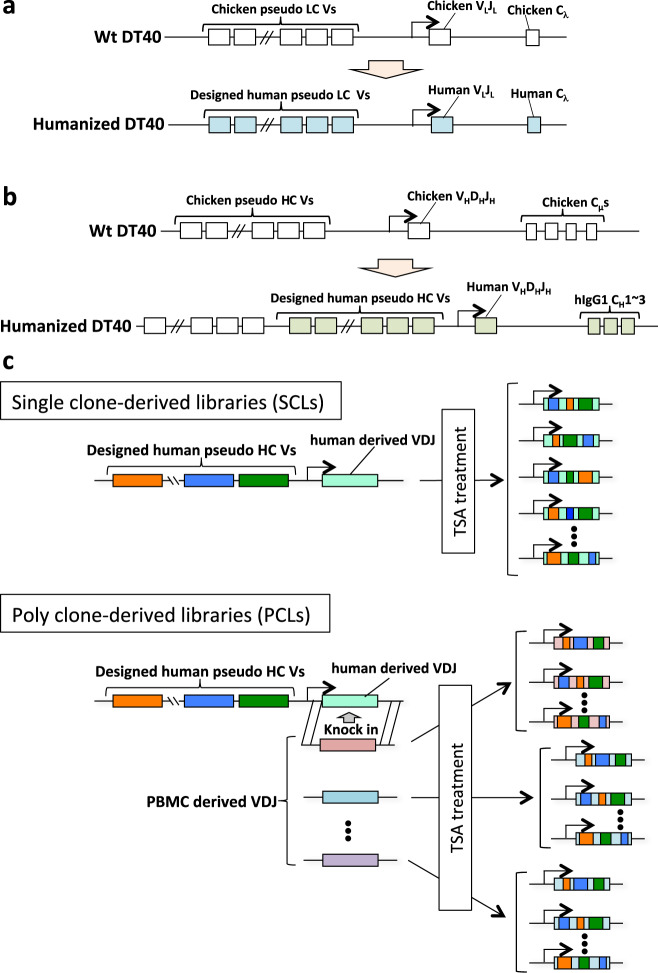
Construction of libraries for the human ADLib system. **a** Replacement and insertion of chicken immunoglobulin LC components (open rectangles) into human counterparts (blue rectangles). The endogenous chicken pseudogenes are replaced with designed pseudogenes. **b** Replacement of the chicken immunoglobulin HC components (open rectangles) with their human counterparts (green rectangles). Designed pseudogenes are inserted downstream of the chicken pseudogene cluster. **c** Two approaches to construct the human ADLib system. In the “single clone-derived library (SCL)” approach, chicken immunoglobulin genes are replaced by human counterparts, and the designed human pseudogenes are inserted upstream of the V_H_D_H_J_H_ region. The immunoglobulin genes of the knocked-in cells are diversified by TSA treatment to enhance GC. In the other approach, “poly-clone-derived libraries (PCLs)”, human PBMC-derived V_H_D_H_J_H_ gene libraries are knocked into the V_H_ region of the cells for PCLs. The obtained knocked-in clones are treated by TSA to expand their diversity in a mixed state

To overcome this drawback, we aimed to develop a human version of the ADLib system that expresses full-length human IgGs for antibody selection. The exons of the HC and LC loci of DT40 cells were replaced by human counterparts, and a variety of designed human pseudogenes were inserted upstream of each functional V region (Fig. 2a, b). We confirmed that by TSA treatment, GC occurred at the humanized immunoglobulin loci of these cells, yielding diversified libraries of human IgGs. From these libraries, clones specific to target antigens, including vascular endothelial growth factor-A (VEGF-A) and tumor necrosis factor α (TNFα), were enriched, with several possessing neutralizing activities. Moreover, mAbs with improved affinities were readily obtained through secondary libraries originating from just 1 or 2 weeks of extended culturing of the parental clones, and their enhanced neutralizing activities correlated well with observed gene conversion events. These results demonstrate that the human ADLib system is a rapid and effective technology platform for the de novo generation and optimization of potential therapeutic human mAb leads for various diseases, including cancer, autoimmune diseases and emerging infectious diseases. Our data also show that molecular libraries can be constructed by introducing exogenous genes into specific loci in DT40 cells, suggesting that the ADLib system can potentially be applied for the molecular evolution of non-antibody proteins for various purposes, including next-generation therapeutics and diagnostics.

## Results

### Design of pseudogenes

**Fig. 3:**
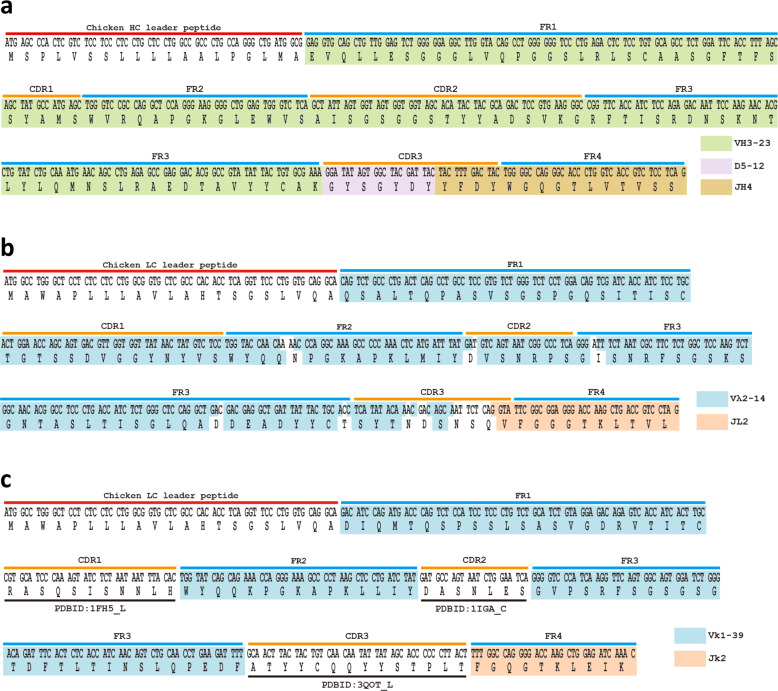
Nucleotide and amino acid sequences of the functional V_H_D_H_J_H_ regions. **a** Nucleotide and amino acid sequences of the functional HC V region. The chicken HC leader sequence, germline VH3-23, D5-12, and JH4 were linked directly without P/N nucleotides. The colored boxes indicate VH3-23 (green), D5-12 (purple) and JH4 (brown). **b** Nucleotide and amino acid sequences of the functional λ LC VJ region. The chicken LC leader sequence and V_L_J_L_ region of Ramos cells were linked. The colored boxes indicate the corresponding germline V (IGLV2-14*01: blue) and J (IGLJ2*01: beige). **c** Nucleotide and amino acid sequences of the functional κ LC V_L_J_L_ region. The frame regions were derived from the sequence obtained by linking Vκ 1-39 and Jκ2. The CDRs were human sequences derived from the database (protein IDs are indicated). The colored boxes indicate the corresponding germline V (Vκ 1-39: blue) and J (Jκ2: beige)

Since GC is a type of homologous recombination, homology of nucleotide sequences between donors (pseudogenes) and recipients (functional V region) is required. In avian immunoglobulin genes, four framework regions (FRs) (FR1–4) are highly homologous among pseudogenes and functional V genes, contributing poorly to the immunoglobulin diversity. Conversely, three CDRs (CDR1–3) are highly diverse and effectively expand the repertoire of immunoglobulin genes (the sequences of the functional V genes and pseudogenes are shown in Fig. 3 and Supplementary Table 1, respectively). We incorporated these structural features into the design of human pseudogenes by inserting CDR1–3 sequences derived from the V region cDNA and germline sequences between conserved FRs (see “METHODS” for details).

### Construction of the human ADLib system

The cells constituting the human ADLib system were prepared by replacing the exons of functional antibody genes of DT40 with their human counterparts as well as inserting a designed human version of the pseudogenes. We attempted two approaches (Fig. 2c), with the first being “single clone-derived libraries (SCLs)”, in which genetically engineered single clones were cultured independently in the presence of TSA as a proof-of-concept experiment to enhance GC and acquire diversity. The other approach was “polyclone-derived libraries (PCLs)”, in which diverse HC V regions derived from human peripheral blood mononuclear cells (PBMCs) were replaced with those of DT40 cells. These cells were further treated with TSA, yielding a mixed state for further diversification to achieve increased coverage of sequence space (Fig. 2c).

To construct SCLs, we genetically engineered DT40 cells by gene targeting^31^ (Supplementary Figs. 1 and 2). With regard to the functional HC locus, the HC constant region (CH1, CH2, CH3, M1, and M2) of human IgG1 (hIgG) was introduced into the endogenous constant region of DT40 with simultaneous deletion of the first exon (Supplementary Fig. 1a, c, and d). Since the ADLib system utilizes membrane-bound and secreted isoforms of antibodies for selection and screening, respectively, cDNA of the secreted version of hIgG’s constant region was fused with the intron and exon containing the hIgG1 transmembrane domain for alternative splicing and expression of both isoforms. The endogenous V_H_, D_H_ and J_H_ regions of DT40 were replaced by the human counterparts derived from human germlines VH3–23, D5–12, and JH4 (three segments were linked directly without P/N nucleotides) (Fig. 3a, Supplementary Fig. 1b, c, and d). Similarly, regarding the functional LC locus, the chicken V_L_, J_L_, and C_L_ regions were also replaced by those of human λ counterparts derived from human Burkitt’s lymphoma Ramos cells (Fig. 3b, Supplementary Fig. 2a, e).

The HC pseudogenes were designed so that the FRs were identical to those of the functional V_H_ region (generated by linking VH3–23, D5–12, and JH4; Fig. 3a), and the human HC CDRs derived from databases were inserted between the FRs. For the LC pseudogenes (Supplementary Table 1), the FRs were identical to those of the Ramos V genes (Fig. 3b), and the human LC λ CDRs were derived from the database (Supplementary Table 1). For each of the LC and HC pseudogene loci, 15 pseudogenes (~7.5 kb; all the designed pseudogenes were placed in the same orientation as the functional V regions) were introduced initially as a proof-of-concept experiment. The chicken LC pseudogene locus was deleted to limit the templates of GC to only the designed human pseudogenes (Supplementary Fig. 2b, e), and the array of 15 designed human LC pseudogenes was subsequently introduced (Supplementary Fig. 2c, e). Meanwhile, the chicken HC pseudo V locus was not deleted because of its size (~80 kb). The array of 15 designed human HC pseudogenes was placed upstream of the V_H_ (Supplementary Fig. 1c, d). To enable the ultimate replacement of the pseudogenes via recombination-mediated cassette exchange (RMCE),^24,32,33^ Cre recombinase recognition sites (loxm3 and loxm7RE) were inserted at the 5′ flanking region of the designed pseudogene cluster (Supplementary Fig. 1c). In each step, the selection marker was eliminated by transient expression of Cre recombinase. The resulting clone was referred to as L15H15.

We analyzed the L15H15 cells by flow cytometry and confirmed the expression of membrane-bound hIgG (Supplementary Fig. 3a, b). Immunoblot analysis of L15H15 culture supernatants also showed the presence of secreted hIgGs (Supplementary Fig. 3c). These results suggest that L15H15 cells can readily be used to generate libraries for mAb selection.

### Preparation of SCLs using L15H15 cells

**Fig. 4:**
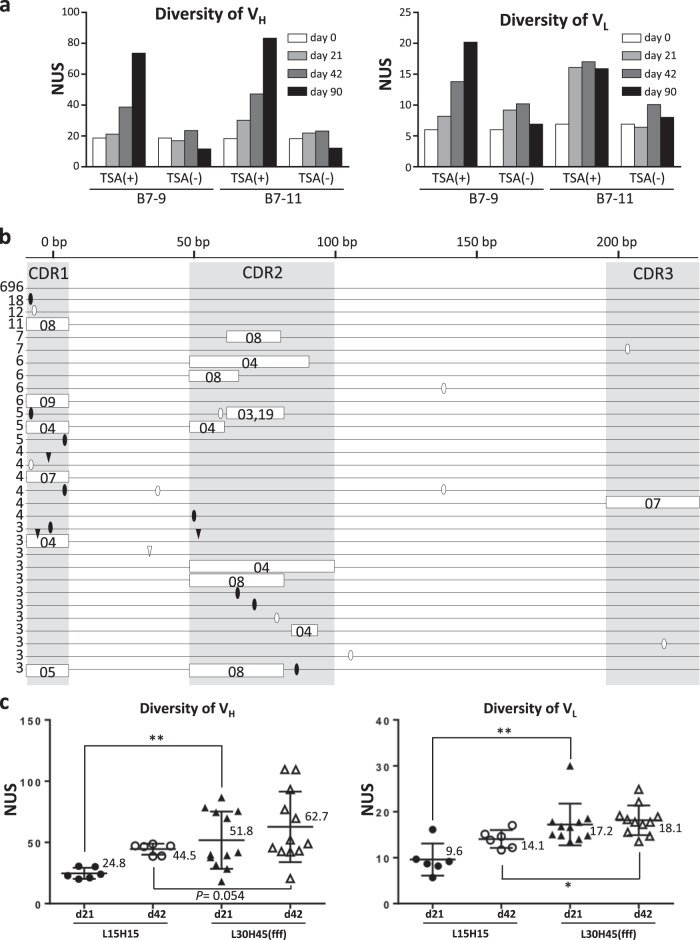
Diversification of SCLs. **a** Diversification of V_H_ and V_L_ sequences in TSA-treated L15H15 cells. Two independent clones (B7-9 and B7-11) were treated for 90 days with or without TSA. The numbers of unique sequences (NUS) in 239 randomly sampled sequences of the V_H_ (left) and V_L_ (right) were examined on days 0, 21, 42, and 90. **b** Schematic V_H_ sequences in L15H15 cells cultured with TSA for 42 days. Horizontal lines represent the sequence reads, and the numbers of identical sequences in the observed whole sequences are shown to the left of the horizontal lines. Open boxes represent putative GC tracts, and the corresponding putative pseudogenes used as templates of GC are described in the abbreviated name (for example, pseudogene “VH08” is described as “08”). Closed ovals show single-nucleotide substitutions that have corresponding sequences in pseudogenes, whereas open ovals are single-nucleotide substitutions that are not attributed to GC. Closed and open triangles represent deletions and insertions, respectively. **c** Comparison of the diversities of L15H15- and L30H45-derived libraries. NUS in 500 randomly selected V_H_ (left) and V_L_ (right) sequences from L15H15-derived (open and closed circles) and L30H45-derived (open and clone triangles) libraries at day 21 (closed circles and triangles) or day 42 (open circles and triangles) of TSA treatment (*n* = 6 for L15H15-derived libraries and *n* = 12 (left) and *n* = 11 (right) for L30H45-derived libraries. Long horizontal bars represent the mean, and their values are shown alongside. Error bars represent ± s.d.). *P < 0.05, **P < 0.01, and ***P < 0.001

**Fig. 5:**
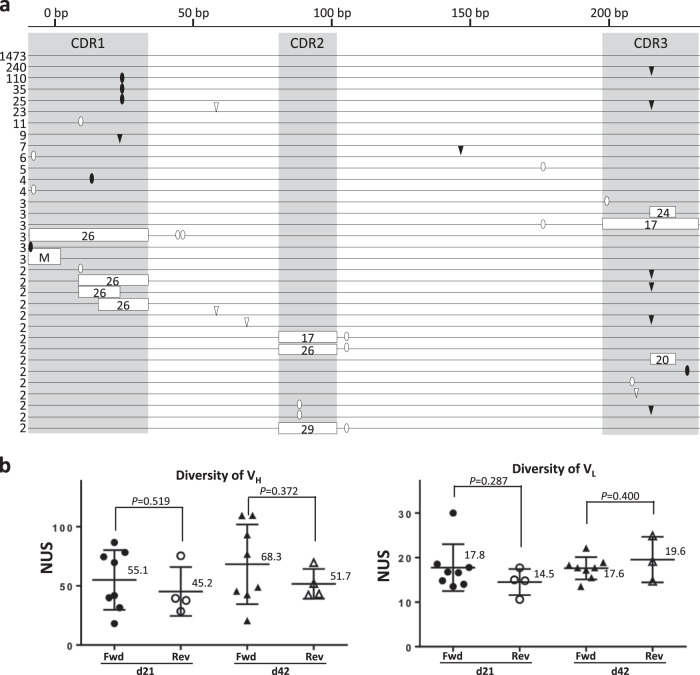
Analysis of diversification of SCLs. **a** Schematic V_L_ sequences in L15H15 cells cultured with TSA for 42 days. Horizontal lines represent the sequence reads, and the numbers of identical sequences in the observed whole sequences are shown on their left. Open boxes represent putative GC tracts, and the corresponding putative pseudogenes used as templates of GC are described in the abbreviated name (for example, pseudogene “VL26” is described as “26”). Note that “multiple” corresponds to any of pVLλ28 and 30. Closed ovals show single-nucleotide substitutions that have corresponding sequences in pseudogenes. Open ovals show single-nucleotide substitutions that are not attributed to GC. Closed triangles and open triangles represent deletions and insertions, respectively. **b** Comparison of the number of unique sequences (NUSs) in 239 randomly selected V_H_ (left) and V_L_ (right) sequences from L30H45(fff) (Fwd)- and L30H45(rrf) (Rev)-derived libraries. Cells treated with TSA for 21 days (d21) and 42 days (d42) were compared (*n* = 8 for Fwd libraries and *n* = 4 for Rev libraries). Long horizontal bars represent the mean, and their values are shown alongside. Error bars represent ±s.d.)

To construct the SCLs, L15H15 cells were cultured in the presence or absence of TSA for 90 days. On days 21, 42, and 90, the sequence diversity of the HC and LC V regions was analyzed by 454GS Junior (Roche)-based deep sequencing. A total of 239 independent sequences were sampled, and the number of unique sequences was counted. The number of unique sequences of the V_H_ and V_L_ regions (hereafter, V_H_D_H_J_H_ and V_L_J_L_ are collectively referred to as the “V_H_ region” and “V_L_ region”, respectively) was markedly increased in the libraries cultured with TSA, whereas those cultured without TSA exhibited little diversification (Fig. 4a). The sequence diversity of the V_H_ region after 42 days of culture was observed mostly in CDRs (Fig. 4b), whereas FRs had few sequence variations. Importantly, the sequence changes in CDRs were attributable to GC events templating one of the designed pseudogenes. We also identified single-nucleotide substitutions that had no corresponding sequence in the pseudogenes, possibly due to somatic hypermutation (SHM). We investigated the usage of pseudogenes after 42 days of culture and found that various pseudogenes were utilized as templates for GC to the V_H_ region (Table 1); on the other hand, pseudogene usage for the V_L_ region was rather limited compared to that for the V_H_ region (Fig. 5a and Table 2).

**Table 1:** Pseudogene usage in GC events occurred at VH of L15H15 strains treated with TSA for 42 days

HC	CDR1						CDR2						CDR3					
	A12-3	A12-4	A12-5	B7-3	B7-9	B7-11	A12-3	A12-4	A12-5	B7-3	B7-9	B7-11	A12-3	A12-4	A12-5	B7-3	B7-9	B7-11
Total reads^a^	717	662	1176	981	994	1174	717	662	1176	981	994	1174	717	662	1176	981	994	1174
V	603	586	991	779	850	994	580	559	904	748	855	945	691	628	1119	894	946	1108
Full match^b^	64	35	101	111	51	99	6	2	25	16	5	23	0	3	9	6	10	5
pVH001	0	0	0	2	0	1	0	0	0	0	0	0	0	0	0	0	0	0
pVH003	1	0	0	1	1	3	0	0	0	1	0	0	0	0	0	0	0	0
pVH005	0	1	0	0	1	3	0	0	0	1	0	0	0	0	2	0	0	0
pVH007	0	1	0	0	0	0	0	0	0	0	0	0	0	0	0	0	0	0
pVH009	3	4	7	1	1	1	0	0	0	0	0	0	0	0	0	0	0	0
pVH011	0	0	0	0	0	0	0	0	0	0	0	0	0	0	1	0	0	0
pVH013	11	3	14	15	11	9	0	0	2	2	0	5	0	0	0	1	0	0
pVH015	25	14	57	29	14	33	0	1	1	2	0	3	0	0	0	0	0	0
pVH017	8	4	6	10	5	9	0	0	0	0	0	1	0	3	5	3	9	4
pVH019	0	1	2	1	0	0	0	1	2	0	1	0	0	0	0	1	0	1
pVH021	3	0	3	3	4	20	0	0	0	0	0	5	0	0	0	0	0	0
pVH023	12	6	10	49	13	18	6	0	19	10	3	8	0	0	0	0	0	0
pVH025	0	0	0	0	0	0	0	0	0	0	0	0	0	0	1	1	0	0
pVH027	0	0	1	0	0	0	0	0	0	0	0	0	0	0	0	0	0	0
pVH029	1	1	1	0	1	2	0	0	1	0	1	1	0	0	0	0	1	0
Partial match^c^	16	11	18	47	23	26	19	12	34	25	19	33	7	9	14	7	5	18

^a^The total number of observed sequences in each library

^b^The number of sequences in which whole pseudogene sequences are copied

^c^The number of sequences not identical to any of initial V sequence or pseudogenes

**Table 2:** Pseudo gene usage in GC events occurred at VL of L15H15 strains treated with TSA for 42 days

LC	CDR1						CDR2						CDR3					
	A12-3	A12-4	A12-5	B7-3	B7-9	B7-11	A12-3	A12-4	A12-5	B7-3	B7-9	B7-11	A12-3	A12-4	A12-5	B7-3	B7-9	B7-11
Total reads^a^	2625	2264	2977	2370	2093	1717	2625	2264	2977	2370	2093	1717	2625	2264	2977	2370	2093	1717
V	2548	2203	2824	2301	1836	1618	2557	2211	2881	2321	2061	1680	2153	1869	2373	2019	1763	1495
Full match^b^	2	2	6	20	6	9	15	13	20	15	12	16	10	6	15	6	5	2
pVL001	0	0	0	0	0	0	0	0	0	0	0	0	1	0	0	0	0	0
pVL003	0	0	1	2	0	0	0	0	1	6	2	1	0	0	1	0	0	0
pVL005	0	0	0	0	0	0	0	0	0	0	0	0	0	0	8	0	0	0
pVL007	0	0	0	7	1	0	0	0	2	6	1	0	0	0	2	0	1	0
pVL009	2	2	3	9	5	8	2	0	4	0	3	10	0	0	0	1	0	0
pVL011	0	0	0	0	0	0	0	0	0	0	0	0	1	0	0	0	0	0
pVL013	0	0	0	0	0	0	7	2	2	0	1	0	1	0	0	0	0	0
pVL015	0	0	0	0	0	0	4	0	0	0	1	1	0	0	0	0	0	0
pVL017	0	0	0	0	0	0	1	11	9	0	2	4	0	0	0	0	0	0
pVL019	0	0	0	0	0	0	0	0	0	0	0	0	0	2	2	0	0	0
pVL021	0	0	0	0	0	0	0	0	0	0	0	0	0	0	0	0	0	0
pVL023	0	0	0	0	0	0	0	0	0	0	0	0	1	2	0	0	0	0
pVL025	0	0	0	2	0	0	0	0	0	2	2	0	3	1	0	4	1	0
pVL027	0	0	2	0	0	1	1	0	2	1	0	0	3	1	2	1	3	2
pVL029	0	0	0	0	0	0	0	0	0	0	0	0	0	0	0	0	0	0
Partial match^c^	17	10	2	3	4	12	3	0	3	9	0	2	2	1	2	0	0	1

^a^The total number of observed sequences in each library

^b^The number of sequence in which whole pseudo gene sequence is copied

^c^The number of sequences not identical to any of initial V sequence or pseudo genes

### Enhanced diversities of libraries by addition of pseudogene copy numbers

Arakawa et al. reported that the reduced copy number of pseudogenes lowers the GC frequency and enhances the frequency of SHM, suggesting that the copy number of pseudogenes is an important determinant of frequent GC.^34^ To test whether the addition of pseudogene copy numbers could improve the diversity of SCLs, we therefore added 15 LC pseudogenes by target integration upstream of the existing LC pseudogenes of L15H15 cells (referred to as clone “L30H15”) (Supplementary Fig. 2d, f). We also introduced 30 HC pseudogenes in the forward orientation upstream of the HC pseudogenes of L30H15 by two rounds of RMCE (15 pseudogenes per round), affording L30H45(fff) (Supplementary Fig. 4a, d). Using these clones, we examined the effects of the copy number of pseudogenes on sequence diversity. After 21 days of TSA treatment, the V_L_ of L30H45(fff) became more diversified than that of the L15H15 libraries, with the effect persisting into day 42, although the increase was not strictly proportional to the copy number of pseudogenes (Fig. 4c). In contrast, although the increased diversity of the V_H_ of L30H45(fff) compared to that of L15H15 showed a significant difference after 21 days of TSA treatment, the values were statistically indistinguishable after 42 days of TSA treatment (Fig. 4c).

According to a previous report, pseudogenes in the reverse orientation relative to the functional V region are preferably utilized as GC templates over those in the forward orientation.^35^ We thus constructed L30H45(rrf) (30 LC and 15 HC pseudogenes were inserted in forward orientation, whereas 30 HC pseudogenes were inserted in reverse) in hope of further boosting the diversity (Supplementary Fig. 4a, b). Contrary to our expectation, the number of unique sequences was comparable between L30H45(fff) and L30H45(rrf) (Fig. 5b).

### Isolation of antigen-specific mAbs from humanized DT40 cells

We then sought to generate mAbs from SCLs using histidine-tagged human semaphorin 3A (hSema3A) fused with alkaline phosphatase (hSema3A-his-AP) as a model antigen. hSema3A is a factor involved in the regulation of neuronal axon guidance, the immune system, angiogenesis and osteoprotection.^36^ We previously succeeded in generating hSema3A-neutralizing mAbs by the original ADLib system.^29,30^ L30H45(fff) cells treated with TSA for 86 days were subjected to mAb selection using hSema3A-his-AP-conjugated magnetic beads, followed by limiting dilution cloning. Three clones specifically reacted with the target (Supplementary Fig. 6a, b), and sequence analysis revealed that these clones had identical HC and LC sequences. In comparison to the original V region, we detected sequence changes in the V_H_ region, possibly due to the transfer of the pseudogenes pVH09 and pVH04 by GC in HC CDR1 (CDR-H1) (Supplementary Fig. 6c). In CDR-H2, partial transfer of pVH04 and pVH08 was observed. The LC sequence was identical to that of the original Ramos sequence. The observation of multiple mutated sequences attributed to GC suggests that the antigen specificity was largely established by GC.

### Construction of PCLs

**Fig. 6:**
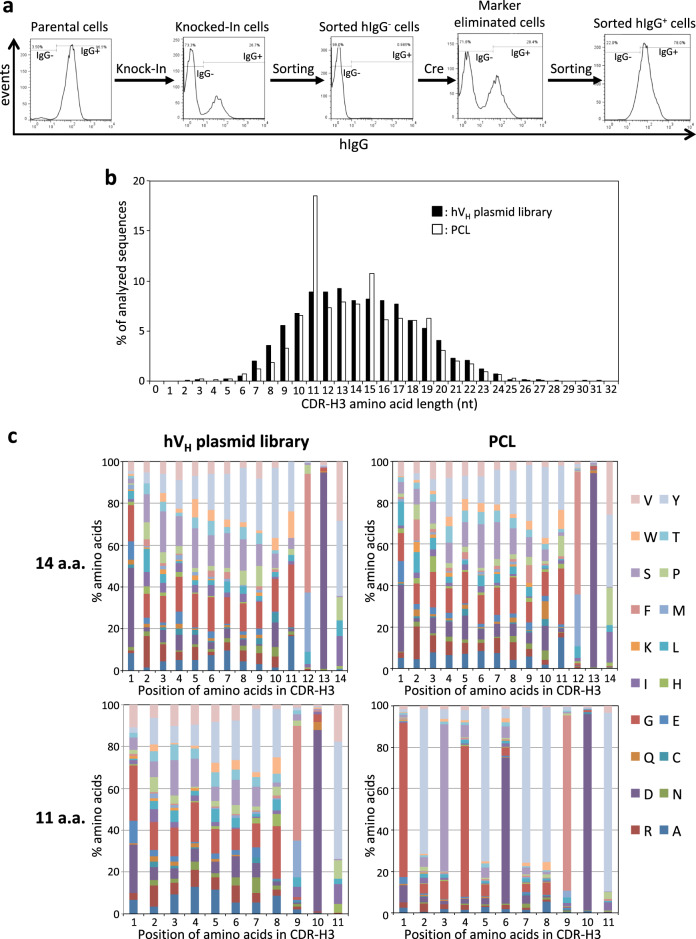
Construction of PCLs. **a** Knock-in of hV_H_ plasmid libraries into humanized DT40 cells. The expression of surface hIgG on the cells in each step was analyzed by flow cytometry. Parental cells (L30H45(fff), leftmost) were transfected with hV_H_ plasmid libraries (second from the left), which led to the loss of cell surface IgG due to insertion of the blasticidin resistance marker between V_H_ and C_H_. The IgG-cells were sorted (middle) to concentrate the knocked-in cells. The knocked-in cells were transfected with the Cre recombinase expression vector (second from the right), and those cells that regained their surface IgG cells were collected by further magnetic sorting (rightmost). **b** Distribution of the amino acid length in CDR-H3 determined by NGS data from plasmid libraries (cDNA: closed bars) and the poly-clone-derived library (PCL: open bars). The distribution pattern of the CDR3-H3 length of the knocked-in library is similar to that of the plasmid library, except that the frequency of the 11-amino-acid sequence is noticeably higher. **c** The distribution of amino acid usage at each position in CDR-H3 was analyzed with respect to 14-residue and 11-residue CDR-H3. The results for the hVH plasmid library (left), PCL (right), 14 amino acids (upper) and 11 amino acids (lower), are shown. The sequence with the most abundant amino acid at each position of the 11-amino-acid-long CDR-H3 of the knocked-in cells was GYSGYDYYFDY. This sequence was identical to the CDR-H3 of the parental cells (L30H45(fff)), possibly derived from nontargeted parental cells during negative IgG selection

The positive results obtained using SCLs led us to explore the feasibility of the second approach, PCLs (Fig. 2c). Diverse V regions were sourced from human cDNAs of healthy donor PBMCs and cloned into an hV_H_ targeting construct using arm sequences identical to those of the V_H_ knock-in vector. The resulting targeting plasmid libraries (hV_H_ libraries) were knocked into the V_H_ region of the L30H45(fff) cells (Fig. 6a). The knocked-in clones were treated with TSA to enhance their diversity in a mixed state. The diversity was later evaluated by comparing hV_H_ sequences between the cDNA libraries and the knocked-in libraries using deep sequencing. The population of cells that underwent targeted integration of exogenous human V_H_ showed sequence diversity in their HC CDR-H3 length (Fig. 6b). The amino acid usage in the 14-amino-acid-long CDR-H3 of the knocked-in cells showed a divergent distribution that was well correlated with the hV_H_ plasmid library (Fig. 6c).

To further expand the diversity of both SCLs and PCLs, an additional 15 V_H_ pseudogenes were integrated by RMCE into L30H45(fff) cells in forward orientation to generate L30H60(ffff) cells (Supplementary Fig. 4c). Moreover, as all the abovementioned humanized DT40 cells contained λ LCs, a κ version of the corresponding LC knock-in construct (consisting of the human κ constant region, κ V_L_ region, and 27 designed human κ V pseudogenes) was established based on L30H60(ffff) to increase the diversity of κ LC (referred to as K27H60(ffff)) (Supplementary Fig. 5a, b). The functional Vκ region was generated by linking the human FRs (corresponding to those of human germline Vκ 1–39 and Jκ2) and the human CDRs derived from the database (Fig. 3c). For the pseudogenes, κ LC CDRs from the database were inserted between the FRs (Supplementary Table 1) identical to those of the functional Vκ. The expression of LC was examined by flow cytometry and immunoblot analysis to confirm the expression of κ LCs (Supplementary Fig. 3a–c). Finally, the hV_H_ plasmid libraries were knocked into L30H45(fff), L30H60(ffff) and K27H60(ffff) cells to create PCLs for subsequent mAb selection.

### Selection of functional mAbs against VEGF

**Fig. 7:**
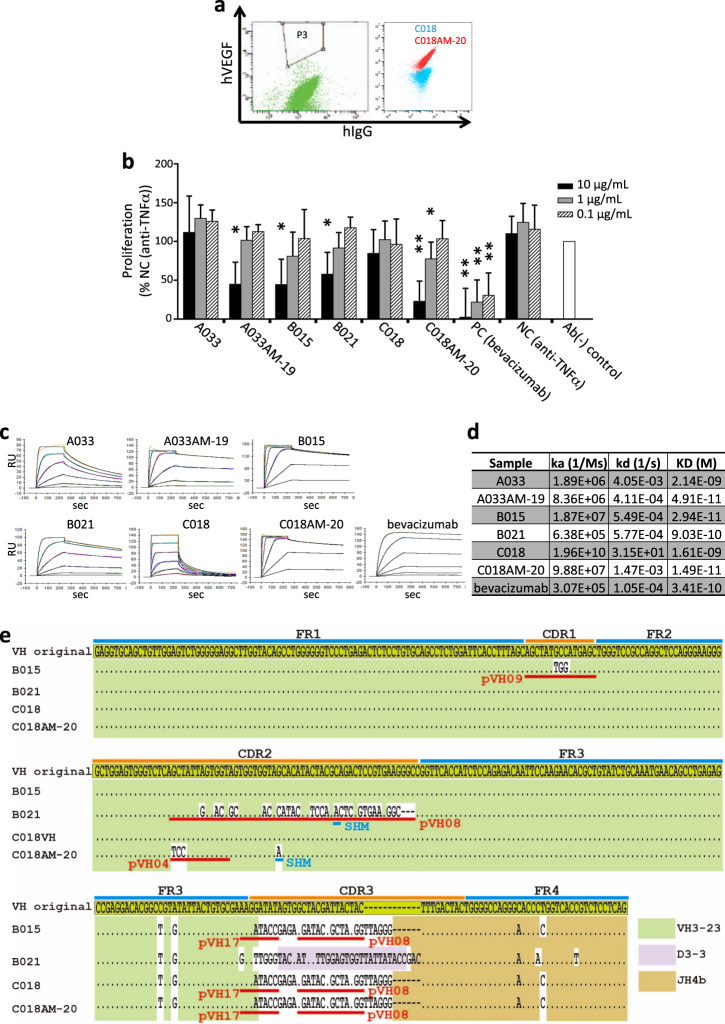
Isolation of VEGF-A-neutralizing mAbs. **a** The sorting experiment to enhance the affinities of the anti-hVEGF cells. The C018 cells cultured without TSA were fluorescently stained with anti-hIgG and hVEGF. The cells showing higher reactivity to the antigen than the major population (gate “P3”) were singly sorted (left). The reactivity of the clones before and after affinity maturation was analyzed by flow cytometry (blue, parental C018; red, C018AM-20, the representative clone after affinity maturation) (right). **b** Bar chart representing the effects of anti-VEGF-A mAbs on the proliferation of HUVECs against the Ab(−) control. VEGF-A and serially diluted anti-hVEGF-A mAbs were preincubated and added to the HUVEC culture. The number of cells was counted after two days. *P* values represent significant differences between the cells cultured in the presence of anti-VEGF mAbs and identical concentrations of the negative control (anti-TNFα) mAb; error bars represent ±s.d. (*n* = 4). *P < 0.05, **P < 0.01, ***P < 0.001. **c** SPR analysis of the anti-hVEGF mAbs. Serially diluted hVEGF-A was applied to the anti-hVEGF-A mAb immobilized on biosensor chips. **d** Summary of antibody binding constants. Association rate (ka) and dissociation rate (kd) constants were determined by SPR analysis. Dissociation constant (KD) values were calculated as kd/ka. **e** The sequences of the anti-hVEGF-A V_H_ regions containing VH3-23-derived sequences. The sequences of B021, B015, C018 and C018AM-20 are compared with the original synthetic VDJ sequence designed for SCLs. The colored boxes indicate the original V genes: V3-23 (green), D3-3 (purple), JH4b (brown). The red and blue horizontal bars represent GC tracts (corresponding pseudogenes are described beside the bars) and SHM, respectively

PCLs produced by the knocked-in cells (L30H45(fff), L30H60(ffff) and K27H60(ffff)) treated with TSA were combined with SCLs (L30H45(fff), L30H60(ffff) and K27H60(ffff)), and the whole mixtures were subjected to mAb selection against human VEGF-A (hVEGF-A). Anti-hVEGF-A clones were enriched by magnetic sorting followed by single-cell sorting. The specificities of the isolated clones were examined by ELISA (Supplementary Fig. 7a). Interestingly, using purified proteins, some of the obtained clones were shown to inhibit the binding of VEGF-A to VEGFR2 (Supplementary Fig. 7b). However, some clones did not show inhibitory activity in a concentration-dependent manner. This suggests that the affinity of the isolated anti-VEGF-A clones might be insufficient for functional activity. Thus, in pursuit of improved affinities of the anti-hVEGF-A clones, we moderately diversified the immunoglobulin genes of anti-hVEGF-A clones (C018 and A033) by prolonged culturing in the absence of TSA. The anti-hVEGF-A clones cultured for 8 days contained a small number of cells reacting with hVEGF-A better than the parental clones under similar surface IgG expression levels. These cells were isolated by a cell sorter and shown to have relatively high reactivity to VEGF-A (Fig. 7a). Correspondingly, C018-AM20 exhibited notable concentration-dependent inhibition of VEGF-A binding to VEGFR2 compared to its parental clone (Supplementary Fig. 7b).

Next, we carried out a cell-based assay^37,38^ to monitor the inhibitory effect of isolated mAbs on the intracellular signal transduction cascades of VEGF. Using human umbilical vein endothelial cells (HUVECs), we showed that several clones possess inhibitory activity against p44/42 MAPK phosphorylation (Supplementary Fig. 7c). Moreover, these mAbs were shown to inhibit the ability of hVEGF-A to stimulate the proliferation of HUVECs (Fig. 7b). SPR experiments revealed that all of the isolated anti-VEGF-A clones had nM-order KD values (Fig. 7c, d). Remarkably, the clones obtained after affinity maturation (A033AM-19 and C018AM-20) displayed ~100-fold higher affinities (Fig. 7c, d: KD = 4.91 × 10^−11^ M and KD = 1.49 × 10^−11^ M, respectively) than the corresponding parental clones (KD = 2.14 × 10^−9^ M and KD = 1.61 × 10^−9^ M, respectively) and exhibited improved antagonistic activity (Fig. 7b, Supplementary Fig. 7b, c), suggesting that the streamlined affinity maturation process contributes to the improvement of the functionalities of mAbs. Strikingly, the increased affinities resulted from a drastic decrease in the dissociation rate constants (Fig. 7d).

Sequence analysis of the HCs revealed that B015, B021, and C018 contain VH3-23-derived sequences (Fig. 7e). Among them, B021 contains a D3-3-derived sequence. Since D3-3 was significantly different from the knocked-in original synthetic VDJ sequence (VH3-23, D5-12, and JH4) designed for the SCL, it was speculated that the sequences were derived from PCLs. The D regions of B015 and C018 could not be identified due to sequence alteration, possibly by GC events, and their J regions were more similar to JH4b than the synthetic VDJ sequence. For the LC, B015, B021, and C018 were κ versions, and various GC tracts were observed (Supplementary Fig. 7d). Interestingly, comparison of the C018 and C018AM-20 HCs revealed that they are different in terms of a pseudogene-derived sequence (pVH04) and a point mutation at CDR2 (Fig. 7e). As their LCs are identical, the GC and SHM likely confer improved affinities. A033 HC harbors VH1-69 (Supplementary Fig. 7e), indicating that it originated from the PCL. The LC of A033 was of the λ version, and corresponding sequences to two pseudogenes were observed (Supplementary Fig. 7f). The LC sequence of A033AM-19 was identical to that of A033, implying that the transfer of pVH09 to A033 CDR-H1 by GC is responsible for the improved affinity of A033AM-19.

### Selection of functional mAbs against TNFα

**Fig. 8:**
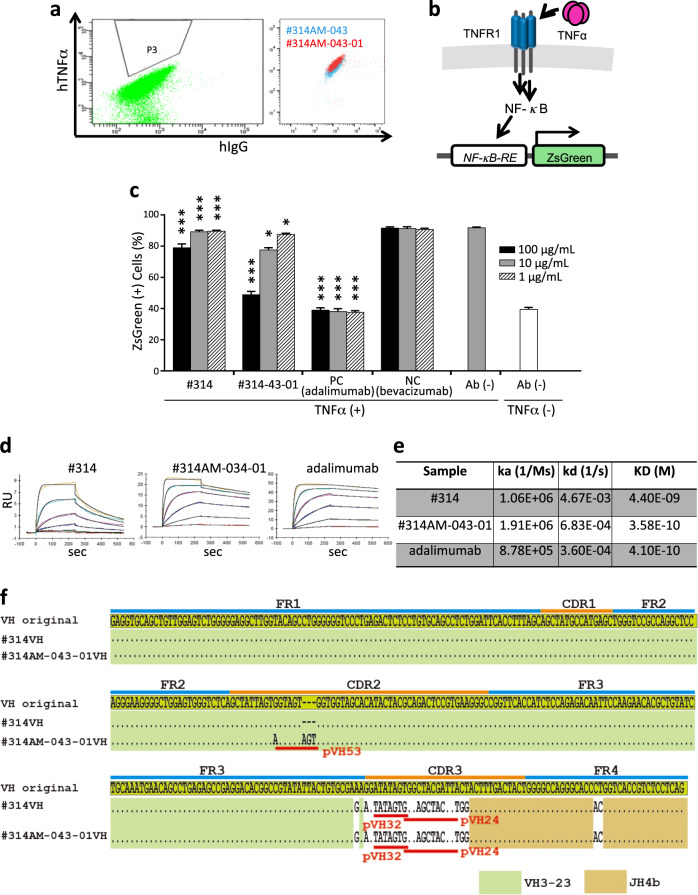
Isolation of the anti-hTNFα-neutralizing mAbs. **a** The sorting experiment for affinity maturation. The #314-043 cells cultured without TSA were stained with anti-hIgG and 10nM hTNFα. The cells showing higher reactivity to antigen than the major population (gate “P3”) were singly sorted (left). The newly isolated and parental clones were analyzed by flow cytometry (right; #314-043 (blue) and #314AM-043-01 (red)). **b** Schematic representation of the principle of the reporter assay to detect NF-κB signaling. The binding of hTNFα to TNFR1 activates the NF-κB signaling pathway, resulting in enhanced expression of ZsGreen. **c** Bar chart representing the neutralizing activities of anti-hTNFα mAbs against ZsGreen expression stimulated by hTNFα. hTNFα and serially diluted anti-hTNFα mAbs were preincubated and added to BaF3 cells carrying the reporter construct followed by counting of ZsGreen+ cells. The results for #314 and #314AM-043-01 are shown. Adalimumab and bevacizumab were used as positive control (PC) and negative control (NC) mAbs, respectively. *P* values represent significant differences between the cells cultured in the presence of anti-TNFα mAbs and identical concentrations of bevacizumab; error bars represent ±s.d. (*n* = 4). *P < 0.05, **P < 0.01, ***P < 0.001. **d** SPR analysis of the anti-TNFα mAbs. Serially diluted purified TNFα was applied to the anti-TNFα mAb immobilized on biosensor chips. **e** Summary of antibody binding constants. The association rate (ka) and dissociation rate (kd) constants of the anti-TNFα mAbs were determined by SPR analysis. Dissociation constant (KD) values were calculated as kd/ka. **f** The sequences of the anti-TNFα V_H_ regions derived before (#314) and after (#314AM-043-01) affinity maturation are compared with the synthetic VDJ

Next, anti-human TNFα (hTNFα) clones were selected from the mixed library by hTNFα-conjugated magnetic beads. The specificities of the candidate clones were analyzed by ELISA (Supplementary Fig. 8a). The clone with the strongest ELISA signal (#314) was further cultured without TSA for 11 days, and a subclone reacting to hTNFα better than #314 was obtained (#314-AM043) (Supplementary Fig. 8b). #314-AM043 was further diversified for an additional 14 days and subjected to a second round of affinity maturation to obtain a clone with even better reactivity (#314AM-043-01) (Fig. 8a). The neutralizing activity of these clones was examined by NF-κB activation.^39^ The reporter construct containing a fluorescent protein (ZsGreen) and an NF-κB response element was stably integrated into BaF3 cells. Binding of TNFα to TNFR1 activates the NF-κB signaling pathway, resulting in upregulated ZsGreen expression (Fig. 8b, c). The #314 clone was shown to have neutralizing activity (Fig. 8c), and undoubtedly, the clone obtained after two rounds of affinity maturation (#314AM-043-01) showed improved functionalities, which was correlated with a one-order improvement in affinity as determined by SPR (Fig. 8d, e).

Sequence analysis of the HC of #314 revealed that it contained VH3-23 (Fig. 8f). Although the original D region could not be identified because of alteration by two GC events, this region might have been derived from PCLs since the J regions are more similar to JH4b than the VDJ sequence designed for SCLs. The sequence corresponding to pseudogene pVH53 was found in the CDR-H2 of #314AM-043-01, indicating that the improved affinity is conferred by GC. The LC of #314 was of the κ version, and moreover, a sequence corresponding to a pseudogene was found in CDR-L1, which could also be attributed to GC (Supplementary Fig. 8c).

## Discussion

In this paper, we describe the development of the human version of the ADLib system by replacing chicken immunoglobulin genes with the corresponding human counterparts and, most importantly, by introducing human pseudogenes. DT40 cells with designed human immunoglobulin loci capable of building unique immunoglobulin repertoires were diversified by TSA treatment to generate cell-based display libraries for the isolation of mAbs with functional activities against various antigens, as exemplified by neutralizing anti-hVEGF-A and anti-hTNFα clones. Furthermore, clones with improved affinities and functionalities were seamlessly obtained after being first isolated by a simple culturing process to moderately diversify the cell population. Taking full advantage of this special property of DT40 cells, the affinity maturation process requires just one to two weeks of culturing followed by cell sorting.

Copious cell-based human mAb display technologies have been reported to date.^7–13^ In most cases, mAbs are generated from predetermined libraries constructed by exogenous introduction of diverse human antibody genes. The ABELmAb library, however, is one of the exceptions.^10^ In this method, antigen-specific clones are generated from fixed libraries, and the subsequent overexpression of activation-induced deaminase (an essential factor of SHM^40^) in these clones introduces SHMs into the immunoglobulin genes, allowing affinity maturation of the isolated clones. Meanwhile, the human ADLib system is unique in that the libraries are actively diversified by GC in addition to SHM, creating an increased capacity of variation.

It has been reported that GC events can be induced among exogenous sequences incorporated into DT40 cells. For example, Kanayama et al. knocked in blue fluorescent protein (BFP) and green fluorescent protein (GFP) genes into the immunoglobulin locus of DT40 and observed GC events between BFP and GFP.^41^ In other reports, Leighton et al. and Schusser et al. replaced the immunoglobulin genes of DT40 with human counterparts, inserted designed human pseudogenes, and observed that GC occurred moderately among V regions and pseudogenes,^42,43^ as a prerequisite proof-of-concept experiment for the establishment of transgenic chickens (OmniChicken) that generate human mAbs in vivo based on GC events among human sequences.^18^ Therefore, the human ADLib system is the first demonstration of the use of GC-based technology for the coupled in vitro generation and affinity maturation of human mAbs.

Both the ADLib system and OmniChicken transgenic animal technology rely on the antibody repertoire exhibited by avian B cells in vitro and in vivo, respectively, for the discovery of mAbs with desired functionalities. Although it is plausible that the DT40 cells constructed by Schusser et al. could provide an in vitro diversified antibody repertoire if treated with TSA, the sequence diversity of the hypothetical libraries might be lower than that of our system given the presence of considerably fewer copy numbers of pseudogenes (<17 copies for HC and <16 copies for LC) being introduced than those in the DT40 cells constituting the human ADLib system.^42,43^ Homologous recombination between the exogenously introduced human V region and pseudogenes has been verified in the B cells of OmniChicken in vivo, yet higher diversity could, in theory, be achieved if a larger number of pseudogenes are integrated. Considering the differences in the number and the adopted sequences of pseudogenes, the human ADLib system and OmniChicken could provide different mAb repertoires, suggesting that the two avian-based systems can be used complementarily.

Transgenic animals, including OmniChicken, have been reported to work well as in vivo methods of mAb generation^1,14–19^; however, difficulties in establishing stable chicken hybridomas necessitate special cloning techniques to retrieve the V region genes from B cells expressing antigen-specific mAbs. Moreover, the HC constant regions of OmniChickens as well as the engineered DT40 cells are in chicken format (IgM, IgY, and IgA), thereby requiring additional genetic engineering processes to obtain mAbs in human IgG format, not to mention the extra work of expression and purification of recombinant antibodies for downstream evaluation. In contrast, activities of mAbs identified by our human ADLib system can be directly evaluated in human IgG format using culture supernatants of DT40. By harnessing the rapid proliferation property of DT40 cells, up to hundreds of micrograms of human IgG can also be easily obtained from the supernatants by affinity purification for analytical characterization or for other assays requiring relatively low protein amounts.

The key features of the human ADLib system for effective antibody generation are (i) the unique diversity generated by GC, (ii) seamless and efficient affinity maturation after initial isolation of a prototype mAb, and (iii) rapidness and low number of manipulation steps required for the isolation and affinity maturation of mAbs. Each of these attributes is expected to greatly facilitate the discovery and optimization of lead therapeutic and diagnostic antibodies for multiple diseases, such as cancer, autoimmune diseases and emerging infectious diseases. Moreover, the ADLib system’s distinctive mechanism for active building of the antibody library provides an alternative to conventional antibody generation methods, enabling the creation of human mAbs with novel activities and potentially better developability. Clearly, our results also indicate that artificial libraries can be constructed in DT40 cells by introducing exogenous genes and their corresponding homologs into immunoglobulin loci, thereby suggesting the possibility of exploiting the ADLib system as an in vitro evolution platform for nonimmunoglobulin proteins. Indeed, in addition to a previous report,^41^ we also succeeded in inducing GC events between two homologous genes encoding fluorescent proteins in DT40 cells and showed that novel chimeras of existing fluorescent proteins can be obtained.^44^ By leveraging the power of GC activity in DT40 cells as the “engine” of diversification in a controlled manner, the ADLib system could hence potentially be applied for directed protein evolution and optimization in various fields beyond biomedical applications, such as in material and agricultural sciences.

## Methods

### Cell culture

DT40 cells were cultured at 39.5 °C in 5% CO_2_ in IMDM containing 10% fetal bovine serum (Access), 1% penicillin/streptomycin, 0.5 μM monothioglycerol and 1% chicken serum (Thermo Fisher) as described.^22,23,26^ For TSA treatment, cells were passaged every day at a concentration of 3 × 10^5^ cells/mL in medium containing 2.5 ng/mL TSA (Fujifilm). HUVECs were cultured in formulated EGM^TM^-2 endothelial growth medium (Lonza) at 37 °C in 5% CO_2_. BaF3 cells were cultured at 37 °C in RPMI 1640 medium containing 1 ng/mL recombinant mouse IL-3 (R&D System), 1% penicillin/streptomycin, and 10% fetal bovine serum (Access).

### Design of the pseudogenes

A pseudogene unit consisted of FR1, CDR1, FR2, CDR2, FR3, CDR3, and FR4, in that order. For the Igλ and Igκ chains, the FRs of all the pseudogenes were based on those of hV_L_, consisting of IGLV2-14*01 and IGLJ2*01, and hV_L_, consisting of Vκ 1-39 and Jκ2, respectively. For hV_H_, FRs obtained by linking human germline VH3-23, D5-12, and JH1 sequences were used. A variety of pairs of CDR1 and CDR2 sequences from Igλ and HC were extracted from the germline V genes found in V-BASE (http://www2.mrc-lmb.cam.ac.uk/vbase/) and human immunoglobulin V genes found in GenBank (http://www.ncbi.nlm.nih.gov/genbank/) (30 pairs for Igλ and 59 pairs for HC). After the sectioning of each sequence according to the Kabat numbering scheme, CDR1 and CDR2 sequences were obtained. Regarding the design of CDR3 sequences, approximately 1000 nonredundant CDR3 sequences were obtained from V-BASE and GenBank. In addition, the hypothetical CDR3 sequences were designed to reflect all combinations of V, D, and J regions. Next, a phylogenetic tree was constructed by the neighbor-joining method using the CDR3 candidate sequences. The 60 individual CDR sequences were selected for HC pseudogene construction. With regard to Igλ, 30 individual CDR sequences were selected for Igλ pseudogene construction. These sequences represent major clusters of the phylogenetic tree. Amino acid lengths also vary. For Igκ pseudogene construction, the κ V genes were obtained from GenBank and sectioned for each CDR. Twenty-seven individual CDR sequences with a Levenshtein distance of 2–7 against the functional κ V gene were selected for each CDR. The median amino acid lengths were 12, 11, and 9 for HC, Igλ, and Igκ, respectively. Levenshtein distance was calculated by R with the Biostrings package (Supplementary Table 1).

### Construction of the V_L_ and constant region knock-in vector

cDNA of Ramos was prepared using SuperScript® III Reverse Transcriptase (Thermo Fisher Scientific), and Ramos λ V region (hV_L_) VL6 (GenBank Z74694.1) was amplified with the primers F1 and R1　(primers are described in Supplementary Table 3). The Ramos Igλ constant region (hCλ) was amplified with the primers F2 and R2. The noncoding 1.5-kb genomic region of DT40 adjacent to and upstream of hV_L_ was amplified with the primers F3 and R3 and referred to as the left arm. The 1.7-kb center arm fragment corresponding to the genomic locus between hV_L_ and hCλ was synthesized. The noncoding 2.0-kb DT40 genomic region adjacent to and downstream of hCλ was synthesized and referred to as the right arm. The hV_L_ and hCλ regions along with the drug resistance genes and flanking lox sequences were placed between homology arms. All the fragments (left arm, hVL, center arm, hCλ and right arm) were assembled into pBluescript SK (-) by the In-Fusion system (Takara Bio). For the κ chain, functional V_L_ was designed based on Vκ1-39 and Jκ2 and synthesized along with the hCκ sequence. The left arm (amplified by F4 and R4), Vκ, blasticidin resistance genes (F5 and R5) and Cκ (F6 and R6) were amplified and fused by assembly PCR. The resulting fragment was digested with XhoI and NotI and cloned into the XhoI and NotI sites of the Igλ knock-in vector.

### Construction of the chicken LC pseudogene knock-out vector

A 2.8-kb DT40 genomic region adjacent to and upstream of the chicken LC pseudogenes was amplified by the primers F7 and R7 and used as the left arm. A 1.9-kb genomic region adjacent to and downstream of the LC pseudogenes was amplified by the primers F8 and R8 and used as the right arm. The left and right arms were assembled into pUC19 by In-Fusion along with the neomycin resistance marker.

### Construction of the LC pseudogene knock-in vector

Each pseudogene unit was linked by a 125-bp linker sequence in which a 95-bp sequence was derived from the intervening region between chicken endogenous pseudogenes ψVL1 and ψVL2 (GCCTGACACACACAGCCCTCCCCAGACTGTGGGATGAGGCCCTGTGCCCGCAGTCACATGTGGAATATCAAGACACACACATCTATGACAATCAC) and a 30-bp index sequence. Arrays of 15 or 30 pseudogenes linked by the linkers were synthesized (Genewiz). Each array was cloned into AgeI and ClaI sites located between the downstream lox sequence and the left arm in the LC chicken pseudogene knock-out construct. For the κ chain, pseudogenes were linked by sequences derived from the chicken genomic region between LC pseudogenes. Linker sequences are shown in Supplementary Table 2.

### Construction of the HC constant region knock-in vector

The human HC constant region sequence was isolated from the hIgG1 genomic sequence (NW_001838121.1) consisting of CH1, hinge, CH2, CH3, and transmembrane domains (hIgG1 M1 and M2). The intron between each constant region was derived from the mouse IgG2a intron. The 4.3-kb DT40 genomic region adjacent to and downstream of the chicken V region was amplified with the primers F9 and R9 and used as a 5′ homology arm (the center arm). The 0.9-kb DT40 genomic region containing chicken C_μ2_ was amplified with the primers F10 and R10 and used as a 3′ homology arm (the right arm). The targeting plasmid was constructed by assembling the left arm, the hIgG1 constant region, the center arm, the neomycin resistance gene under the control of IRES and the right arm in pBluescript SK(-).

### Construction of the V_H_ knock-in vector

Human hV_H_ was designed by linking human germline VH3-23 and synthetic VDJ sequences consisting of D5-12 and JH4. The 1.4-kb DT40 genomic region adjacent to and upstream of the chicken signal sequence of V_H_ was amplified with the primers F11 and R11 and used as the left arm. The 4.4-kb DT40 genomic region adjacent to and downstream of chicken V_H_ was amplified with　the primers F12 and R12 and used as the right arm. Along with the blasticidin resistance gene, the left arm, hV_H_ and the right arm were cloned into pBluescript SK(-) using the In-Fusion system to construct the hV_H_ knock-in vector.

### Construction of the HC pseudogene knock-in vector

A stretch of 15 pseudogenes (pVH01 to pVH15) was linked to a 125-bp linker sequence as described in the LC pseudogene design. For the knock-in vector of the first 15 pseudogene arrays, the 1.8-kb DT40 genomic region immediately upstream of the left arm of the V_H_ knock-in vector described above was used as the 5′ homology arm (left arm). The right arm was amplified with the primers F11 and R11. The SV40 promoter, along with the neomycin resistance gene in reverse orientation flanked by lox511 sequences, was placed upstream of the 15 pseudogene array. Downstream, the SV40 promoter along with duplicated terminators was placed for the screening of targeted clones with sequential RMCE. The loxm3 sequence (ATAACTTCGTATATGGTATTATATACGAAGTTAT) upstream of the terminators and the loxm7RE sequence (ATAACTTCGTATATTCTATCTTATACGAACGGTA) downstream of the terminators were incorporated for RMCE. The targeting vector was constructed in pUC19 in the following order: the left arm, lox511RE sequence, SV40-neomycin resistance gene in reverse orientation, lox511LE sequence, SV40 promoter, loxm3 reverse sequence, duplicated terminators, loxm7RE sequence, array of 15 pseudogenes, right arm and human HC V region.

### Construction of the RMCE donor vector

For introduction of the pseudogene array by the first and third rounds of RMCE, the donor vector was designed in the following order: the loxm3 reverse sequence, neomycin resistance gene without the SV40 promoter, duplicated terminators, loxPLE sequence (ATAACTTCGTATAATGTATGCTATACGAACGGTA), array of 15 pseudogenes, and loxm7LE sequence (TACCGTTCGTATATTCTATCTTATACGAAGTTAT). The donor vector for the second round of RMCE was assembled in the following order: the loxm3 reverse sequence, blasticidin resistance gene without the SV40 promoter, duplicated terminators, loxm7RE sequence (ATAACTTCGTATATTCTATCTTATACGAACGGTA), 15 pseudogene array, and loxPRE sequence (TACCGTTCGTATAATGTATGCTATACGAAGTTAT). All the components were cloned into pUC57. The 15 pseudogenes (pVH16 to pVH30) for RMCE were linked to a 125-bp linker sequence as described in the LC pseudogene design. The 15 pseudogenes (pVH31 to pVH45) for the 2nd round of RMCE were linked with 100-bp sequences derived from the chicken genomic region between HC pseudogenes. The 15 pseudogenes (pVH46 to pVH60) for the 3rd round of RMCE were linked to linker sequences as described in the LC pseudogene design except that 30-bp index sequences were used only for pVH48, 50, 52, 53, 55, 57, and 59. Each linker sequence is shown in Supplementary Table 2.

### Construction of the knock-in library of the human V_H_ region and the generation of the PCL

The germline-derived divergent hV_H_ harboring CDR3 was prepared from normal human peripheral blood CD19 + B-cell cDNA (ALLCELLS) using degenerate primers that annealed to FR3 (F13) and FR4 (R13). The PCR fragment harboring CDRs 1 and 2 was prepared using another primer set that annealed to FR1 (F14) and FR3 (R14). These PCR fragments were assembled by PCR using primers (F15) and (R15) and then cloned into EcoRV and NdeI sites in the hV_H_ targeting vector with the CAG promoter-blasticidin resistance gene flanked by Vlox sequences (VloxM1; TCAATTTCCGAGAATGACAGTTCTCGGAAATTGA). The resultant construct was transformed into *E. coli*, the colonies were harvested, and plasmids were purified. The pooled library plasmids were linearized by PciI and introduced into the cell by electroporation. The hIgG1-negative cells were purified by a MACS column (Miltenyi), and the recovered cells were expanded. The VCre recombinase expression vectors were transiently transfected, and the resultant cells were subjected to MACS purification to concentrate the hIgG1-positive cells. The obtained cells were diversified by TSA treatment for 21 or 42 days.

### DNA Transfection

The knock-in vectors were transfected by GenePulser (Bio-Rad Laboratories) as previously described.^24^ Vectors were linearized by NotI, except that SalI and ScaI were used for HC constant region knock-in and the hV_H_ knock-in vector, respectively. RMCE donor vectors were transfected using Nucleofector 2b (Lonza) and Cell Line Nucleofector Kit T (Lonza). A total of 1 × 10^7^ cells were collected and transfected with 8 μg of DNA mixture (7 μg of RMCE construct and 1 μg of Cre recombinase expression vector) with the Nucleofector 2b (Lonza) optimized transfection program B-023.

### Genotyping PCR

Genetic engineering by knock-in and RMCE was examined by PCR using PrimeSTAR^®^ GXL DNA Polymerase (Takara Bio). The primer pairs are shown in Supplementary Table 3.

### Flow cytometry

Cells (5 × 10^5^) were washed twice with FCM buffer (PBS containing 0.05% bovine (BSA) serum albumin and 2 mM EDTA) and stained with antibodies for 30 min at 4 °C. The antibodies used were R-PE conjugated goat anti-human IgG (gamma chain specific) (SouthernBiotech), R-PE conjugated goat anti-human Igλ (SouthernBiotech), and FITC conjugated goat anti-chicken IgM (Bethyl Laboratories). After washing with FCM buffer three times, cells were suspended in FCM buffer and analyzed by FACSCanto II (Becton, Dickinson and Company). The data were analyzed by FlowJo software (Becton, Dickinson and Company).

### Next-generation sequence

Genomic DNA was isolated from 1 × 10^6^ cells after TSA treatment. The V_L_ regions were amplified by PCR using sense primer (CGTATCGCCTCCCTCGCGCCATCAGNNNNNNNNNNCAGGTTCCCTGGTGCAGGC, where N indicates index sequences) and antisense primer (CTATGCGCCTTGCCAGCCCGCTCAGGCTTGGTCCCTCCGCCGAA), and V_H_ regions were amplified with sense primer (CTATGCGCCTTGCCAGCCCGCTCAGTCCGTCAGCGCTCTCT) and antisense primer (CGTATCGCCTCCCTCGCGCCATCAGNNNNNNNNNNTGGGGGGGGTTCATATGAAG, where N indicates index sequences). PCR products were purified and analyzed using a 454 GS-junior system (Roche Diagnostics). The sequence reads with lengths less than 250 bp between the first read and the sequence position with quality values <15 were filtered out from further analysis, and each sequence was sectioned into the immunoglobulin framework and CDR according to the Kabat numbering scheme. To evaluate the number of unique sequences (NUS), we repeated 10 attempts of the following cycles: (i) a certain number of read extractions, (ii) determination of the FR/CDR, and (iii) counting of unique sequence reads. The average of the 10 attempts was determined as NUS.

### Antigen preparation

The Flag-tagged proteins (FLAG-hTNFα, FLAG-hHer2, and FLAG-hVEGF-A) were transiently expressed in FreeStyle293-F cells (Thermo Fisher Scientific) and purified by anti-FLAG M2 affinity chromatography (Sigma) followed by gel filtration chromatography (GE Healthcare). hSema3A-his-AP was stably expressed in HEK293 cells (a kind gift from Dr. Yoshio Goshima) and purified by a HisTrap Excel column (GE Healthcare) followed by gel filtration chromatography.

### Isolation of antigen-specific cells from the ADLib libraries

The antigen-specific clones were isolated as described with some modifications.^22,23^ The purified proteins were immobilized onto Dynabeads M-270 Epoxy, Dynabeads M-270 Carboxylic acid and Dynabeads Isolation and Pulldown (Thermo Fisher Scientific). The library cells were seeded at a concentration of 1.5 × 10^7^ cells/40 mL and cultured for 24 h. Cells were harvested, washed, and resuspended in 950 μL of selection buffer (PBS supplemented with 1% BSA), and antigen-immobilized beads were added and incubated at 4 °C for 30 min. The beads were washed out, and the isolated cells were seeded onto 96-well plates and incubated. After 1 week of culturing, the antigen-specific clones were screened by ELISA and subcloned by limiting dilution. Alternatively, cells were isolated by single-cell sorting using a FACS Aria Fusion (Becton, Dickinson and Company). The cloned cells were subjected to a second screening by ELISA.

### Immunoblotting

Immunoblotting was carried out as previously described^24^ with some modifications. The cells were harvested, lysed by sample buffer containing 2-mercaptoethanol and separated by SDS-PAGE using a 4–20% gradient gel. Blotting was carried out using an iBlot 2 Gel Transfer Device and iBind Western Device (Thermo Fisher Scientific). Human immunoglobulin HC, Igλ, Igκ, and chicken IgM were detected by HRP-conjugated goat anti-hIgG-Fc (Bethyl), anti-human Igλ (Southern Biotech), anti-human Igκ (Southern Biotech) and anti-chicken IgM (Bethyl), respectively. ECL western blotting detection reagents (GE Healthcare) and LAS-4000 Mini (Fujifilm) were used for signal detection.

### ELISA

ELISA was carried out as previously described^24^ with some modifications. Antigens were immobilized onto MaxiSorp 384-well plates at 62.5 ng/well at 4 °C overnight and then blocked by blocking buffer (PBS containing 1% BSA). After washing, the culture supernatants were added to the wells and incubated for 1 h at room temperature. Mouse anti-hIgG-Fc HRP conjugated (Southern Biotech) was added to the wells after washing and incubated for 45 min at room temperature. The assay was developed with TMB (Dako), the reaction was stopped with 1 N sulfuric acid, and the absorbance was read at 450 nm.

### Affinity maturation

The antigen-specific clones were cultured in medium without TSA for one week or two weeks. For negative staining, 5.0 × 10^6^ cells were first stained with 100 nM biotinylated Her2 in FCM buffer and further stained with AlexaFluor 488-labeled streptavidin (Thermo Fisher). The cells were harvested and subsequently incubated with 10 nM biotinylated target antigens followed by staining with AlexaFluor 647-labeled streptavidin (Thermo Fisher) and PE-conjugated anti-hIgG (Southern Biotech). The staining conditions were identical to those of FCM. The cells showing high specificities for the antigens at given IgG expression levels were sorted by FACS Aria Fusion (Becton, Dickinson and Company) into 96-well plates.

### hVEGF competition assay

The competition assay was carried out by ELISA with some modifications. Recombinant human VEGF R2/KDR Fc chimera protein (R&D Systems) was immobilized onto MaxiSorp 384-well plates at 62.5 ng/well at 4 °C overnight. The plate was washed with PBS containing 0.05% Tween 20 and soaked with blocking buffer at room temperature for 1 h. During the incubation, the competition reaction mixture of human VEGFR2 (final concentration 20 ng/mL) and the anti-VEGF-A-IgG1 antibody clone (final concentration 0.1, 1, or 10 μg/mL) was preincubated for 30 min at room temperature. Following the removal of blocking buffer and washing, the competition reaction mixture was added into 96-well plates and incubated for 30 min at room temperature. After washing, signals were detected by an HRP-conjugated anti-FLAG M2 antibody (Sigma).

### Phosphorylation inhibition assay

The inhibition of MAPK phosphorylation by anti-VEGF-A mAbs was examined as previously described^37^ using the PathScan Phospho-p44/ 42 MAPK (Thr202/ Tyr204) Sandwich ELISA Kit (Cell Signaling). Pooled 2.5 × 10^5^ HUVECs were seeded into six-well plates and cultured overnight. The medium was removed and washed with 2 mL of Ham’s F-12K containing 1% BSA. The cells were incubated for 6 h for starvation. An aliquot of 110 μL of recombinant hVEGF165 (Pepro Tech) diluted with medium (25 nM) and an equal volume of various anti-VEGF mAbs were mixed and incubated for 30 min at room temperature. Two hundred microliters of the mixture was added to the culture and incubated at 37 °C for 5 min. The plates were then incubated on ice, and the medium was removed. Finally, the cells were washed with ice-cold PBS and lysed, followed by analysis by sandwich ELISA.

### Cell proliferation assay

The inhibition of cell proliferation by anti-VEGF-A mAbs was examined as previously described.^38^ HUVECs were seeded at 2.0 × 10^3^ cells/well in 96-well plates and incubated at 37 °C overnight. On the next day, recombinant human VEGF165 was reacted with serially diluted anti-hVEGF-A-hIgG1 mAbs in medium (VEGF165: 20 ng/mL and anti-hVEGF-A-hIgG1 mAbs: 10, 1, or 0.1 μg/mL) at 37 °C for 1 h before being added to HUVEC culture plates, and then, the cells were further cultured for an additional 2 days in the medium. The viability of the cells was examined with a CellTiter-Glo Luminescent Cell Viability Assay (Promega).

### NF-κB reporter gene assay

The inhibitory activity of anti-TNFα mAbs was examined by a reporter assay essentially as previously described.^39^ An aliquot of 24 pM hTNFα was mixed with serially diluted purified anti-hTNFα mAbs in the medium and incubated for 30 min at room temperature. The mixtures were added to 1 × 10^4^ BaF3 cells carrying the NF-κB reporter construct (Fig. 8b) in 96-well plates and cultured for 4 h. The expression level of ZsGreen was examined by FACSCanto II.

### SPR analysis

SPR analysis was performed as previously described^24^ with some modifications using a Biacore T200 (GE Healthcare). Anti-hIgG capture antibodies (GE Healthcare) were immobilized onto the surface of a CM5 sensor chip via amine coupling. The purified antigens serially diluted with HBS-EP + (100, 33.3, 11.1, 3.7, 1.23, and 0.412 nM) were injected for 240 s at a flow rate of 30 µL/min followed by 300 s of dissociation. Data were fitted with Biacore T200 evaluation software using a 1:1 Langmuir binding model with mass transfer.

### Statistical analysis

Statistical calculations were performed with Microsoft Excel and are described in the relevant figure legends. Differences were compared using a one-tailed *t*-test.

## Supplementary information

Supplementary Fig.1, Supplementary Fig.2, Supplementary Fig.3, Supplementary Fig.4, Supplementary Fig. 5, Supplementary Fig.6, Supplementary Fig. 7 and Supplementary Fig.8

Supplementary Table 1

Supplementary Table 2

Supplementary Table 3
