# Supplementary material for: Streamlined human antibody generation and optimization by exploiting designed immunoglobulin loci in a B cell line
Source: Cell Mol Immunol. 2020 May 26;18(6):1545–61. doi: 10.1038/s41423-020-0440-9 (PMC8166883; doi:10.1038/s41423-020-0440-9)

Supplementary Figure Legends

Supplementary Fig. 1: Genetic engineering of the immunoglobulin HC locus of DT40. **a**, Knock-in of hIgG1 cDNA to chicken HC constant region. The knock-in plasmid was designed so as to disrupt the chicken $C_{\mu 1}$ exon and insert hIgG1 cDNA and the transmembrane domain exon (M1, M2), along with the human derived introns. IRES-driven neomycin resistance gene (Neo) flanked by loxPRE and loxPLE were placed. The selection marker was excised by Cre recombinase transient expression. The blue double headed arrow indicates the amplified regions for genotyping PCR shown in **d**. **b**, Replacement of the chicken $V_HD_HJ_H$ gene to human sequence. Human $V_HD_HJ_H$ sequence was knocked-in to the chicken HC. Blasticidin resistance gene (Bsr), flanked by loxPRE and loxPLE was placed downstream of human $V_HD_HJ_H$. **c**, Insertion of the 15 designed human pseudo HC $V_HD_HJ_H$ genes. Neomycin resistance marker flanked by lox511RE and Lox511LE was placed in the upstream of the designed pseudogenes. Although each pseudogene contains designed V, D and J gene sequences, it is simply referred to as "pseudogene". For the additional insertion of the designed pseudogenes by RMCE, SV40 promoter (SV40P), loxm3 and loxm7RE were integrated. **d**, Genotyping PCR for the HCs of wild type DT40 (wt DT40) and L15H15 cells. Four pairs of primers were used to confirm the knock-in of the designed pseudogenes (H1), human V_H (H0 and H2), and human HC constant region (H3) which are indicated in **a** and **c**. Primers for each genotyping PCR experiment are shown in Supplementary Table 3.

Supplementary Fig. 2: Genetic engineering of the immunoglobulin LC locus of DT40. **a**, Replacement of the chicken V_LJ_L and constant region to Ramos derived sequences. DT40 have two LC alleles (*i.e.* VJ rearranged and unrearranged alleles). Human genes were knocked-in to the rearranged allele. The blue double headed arrows indicate the regions amplified by PCR in **e**. **b**, Deletion of the endogenous chicken pseudogenes. The upstream and downstream regions of chicken pseudogene cluster were used as the arms of the knockout vector. **c**, Introduction of the designed human pseudo V_L genes. 15 designed pseudo Vs (pink rectangles) were knocked-in to the upstream of

the human V_LJ_L. **d**, Introduction of the additional designed human pseudogenes. Additional 15 designed pseudogenes (blue rectangles) were knocked-in to the region between the human V_LJ_L and the previously knoked-in designed pseudogenes. **e**, Genotyping PCR for the LC of wt DT40 and L15H15 cells. Four primer sets in **a** and **c** were used to confirm the knock-in of the designed pseudogenes (L1), human V_L (L0 and L2), and C_λ (L3). With L0, two bands corresponding two alleles are amplified for wt DT40 (rearranged: 464 bp, unrearranged: 2779 bp). **f**, Genotyping PCR for the LC of L15H15, L30H45(fff) and L30H45(rrf). Four primer pairs in **c** and **d** were used to confirm the knock-in of the designed pseudogenes (L1), human V_L and C_λ (L3), and the additional pseudogenes (L4 and L5).

Supplementary Fig. 3: Expression of surface and secreted hIgGs in the humanized DT40 cells. **a**, The expression of cell surface antibodies in L15H15, K27H60(ffff), wt DT40 (IgM-) and wt DT40 (IgM+) cells was analyzed by flow cytometer, using anti-human IgG and anti-chicken Ig M antibodies. **b**, The expression of LC in L15H15 (top row), K27H60(ffff) (second row from the top), wt DT40(IgM-) (third row from the top) and wt DT40(IgM+) (bottom row) cells was analyzed by flow cytometer using anti-human Igλ chain (left column), anti-human Igκ chain (middle column) and anti-chicken Igλ chain (right column) antibodies, respectively. **c**, The expression of secreted antibodies in L15H15, K27H60(ffff) and wt DT40(IgM+) cells analyzed by immunoblot. Anti-human hIgγ Fc (leftmost), anti-chicken IgM (second from the left), anti-human Igλ (third from the left) and anti-human Igκ antibodies (rightmost) were used as primary antibodies and detected by HRP-conjugated secondary antibodies.

Supplementary Fig. 4: Introduction of the additional designed human HC pseudogenes by RMCE. **a**, Introduction of the 30 HC pseudogenes by RMCE. A stretch of 15 pseudogenes (dark red rectangles) were inserted to loxm3 and loxm7RE sites by first round of RMCE in the identical orientation to that of V_H of construct H30(ff) cells, using neomycin as the selection scheme. In the second round RMCE, additional 15

pseudogenes (green rectangles) were introduced to loxm3 and loxPLE sites in the identical orientation to that of V_H of construct H45(fff) cells, using blasticidin selection scheme. The red arrows represent the orientation of the pseudogenes. **b**, Introduction of the additional 30 designed human pseudogenes by RMCE in reverse orientation. Schematic map after two rounds of RMCE is shown. **c**, Introduction of the additional pseudogenes by a third round of RMCE. Additional 15 pseudogenes (blue rectangles) were inserted to H45(fff) cells by RMCE in forward orientation. The schematic map after RMCE is shown. **d**, Genotyping PCR to confirm the introduction of additional genes to HC to construct L30H45(fff) (left), L30H45(rrf) (middle) and L30H60(ffff) (right). The regions indicated by blue double headed arrows in **a**, **b**, **c** were amplified by PCR shown. Although the predicted size amplified by H10 primer pair using L30H60(ffff) is 2417bp, the actual band size was about 800bp, possibly caused by the unexpected recombination between lox511RE/LE remnant and loxPLE by Cre recombinase.

Supplementary Fig. 5: Introduction of the Igκ and κ version of designed human LC pseudogenes. a, Replacement of the human Igλ and designed human LC λ pseudo Vs to κ version. The knock-in construct harboring human Vκ, Jκ, Cκ and κ version of 27 designed V genes (pseudo **κ** Vs) were integrated into the immunoglobulin LC locus of L30H60(ffff) cells. The upstream and downstream regions of L30H30 LC pseudogenes were used as left and right arms of knock-in vector respectively. To make sure VκJκ, Cκ and pseudo **κ** Vs were knocked-in properly, two selection markers (Neo and Bsr) were used. The blue double headed arrows indicate the regions amplified by PCR shown in **b**. **b**, The results of genotyping PCR for the L30H60(ffff) and K27H60(ffff) cells. Eight pairs of primers (L1, L3, L4, L5, L6, L7, L8 and L9) described in **a** were used to confirm the knock-in of the VκJκ, Cκ and pseudo **κ** Vs. The position of the primers and the expected sizes of the amplicons are shown in **a**.

Supplementary Fig. 6: Isolation of the anti-hSema3A-his-AP from SCLs by the ADLib system. a, ELISA to screen the hSema3A-his-AP

specific clones after the selection by magnetic beads. His-Ubiquitin, ovalbumin (OVA) and streptavidin (SA) were used as negative control antigens. PlexinA4, which is known to bind to hSema3A, was used as a positive control. The results of the representative 21 clones are shown. hSema3A-his-AP specific clone (#183) is indicated by a red arrow. **b**, The specificities of the screened clones confirmed by another ELISA. The clones obtained after initial screening ELISA (described in **a**) were subcloned by limiting dilution and subjected to ELISA. Alkaline phosphatase (AP), His-Ubiquitin, OVA, and SA were used as negative control antigens. **c**, Sequence of the anti-Sema3A-his-AP clone V_H region. The V_H region of the obtained clones was compared with the original V_H sequence of SCL. The red horizontal lines represent GC tracts (corresponding pseudogenes are described on their sides).

Supplementary Fig. 7: Selection of the functional mAbs against VEGF. **a**, Specificities of the anti-hVEGF-A candidates by ELISA. FLAG-tagged human HER2 (hHer2-FLAG) and streptavidin were used as negative control antigens. The red arrows indicate the antigen-specific clones. **b**, Bar chart representing the results of solid phase competitive binding assay. The inhibition of the binding of hVEGF-A to VEGFR2 by the anti-hVEGF-A mAbs was examined. 10 ng/mL of FLAG-tagged hVEGF-A and serially diluted (0.1, 1, 10 μg/mL) anti-hVEGF-A mAbs were pre-incubated and added to the immunoplate coated with hVEGFR2. Signals were detected by anti-FLAG antibody. Bevacizumab and anti-TNFα mAb were used as positive and negative controls, respectively. In **b** and **c**, *P* values represent statistical differences between the samples treated with anti-VEGF mAbs and the identical concentrations of negative control (anti-TNFα) mAb; error bars represent ±s.d. (n=3). $^{*}P<0.05$, $^{**}P<0.01$, $^{***}P<0.001$. **c**, Bar chart representing the inhibition of p44/42 MAPK phosphorylation by anti-VEGF-A mAbs. VEGF-A and serially diluted (1, 10 μg/mL) anti-hVEGF-A mAbs were pre-incubated and added to HUVEC culture. P44/42 MAPK phosphorylation was analyzed by sandwich ELISA using anti-p44/42 MAPK and anti-phosphorylated-p44/42 MAPK antibodies.

d, The sequences of anti-hVEGF-A V_L regions (B021, B015, C018 and C018AM-20) compared with the original knocked-in sequence. **e**, Comparison of anti-hVEGF-A V_H regions. The sequences derived from before (A033) and after (A033AM-19) affinity maturation were compared. **f**, The sequence of the anti-hVEGF-A clone V_L region. V_L of A033 and A033AM-19 were compared with the original V_L of SCL.

Supplementary Fig. 8: Selection of the functional mAbs against TNFα. **a**, ELISA analysis to confirm the specificities of the anti-TNFα candidate clones. FLAG tagged human HER2 protein (hHer2-FLAG) and streptavidin were used as negative control antigens. The antigen-specific clones are indicated by red arrows. **b**, Clone #314 was cultured and stained with 1 nM of hTNFα. The newly isolated and parental clones were analyzed by flow cytometer (right; #314AM (blue) and #314AM-043 (red)). **c**, The sequences of the anti-TNFα V_L regions derived from before (#314) and after (#314AM-043-01) affinity maturation were compared.

Supplementary Fig. 1

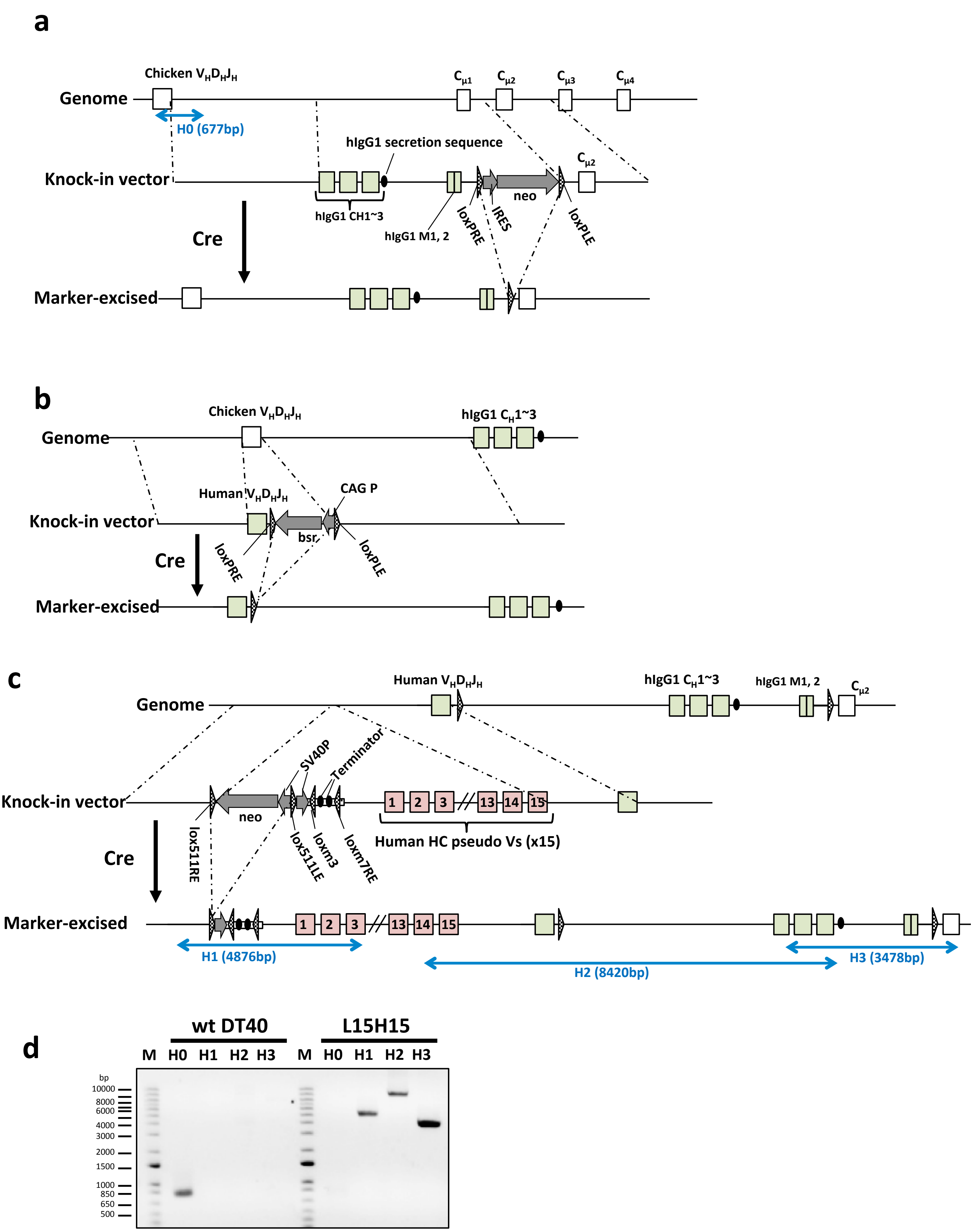

Supplementary Fig. 2

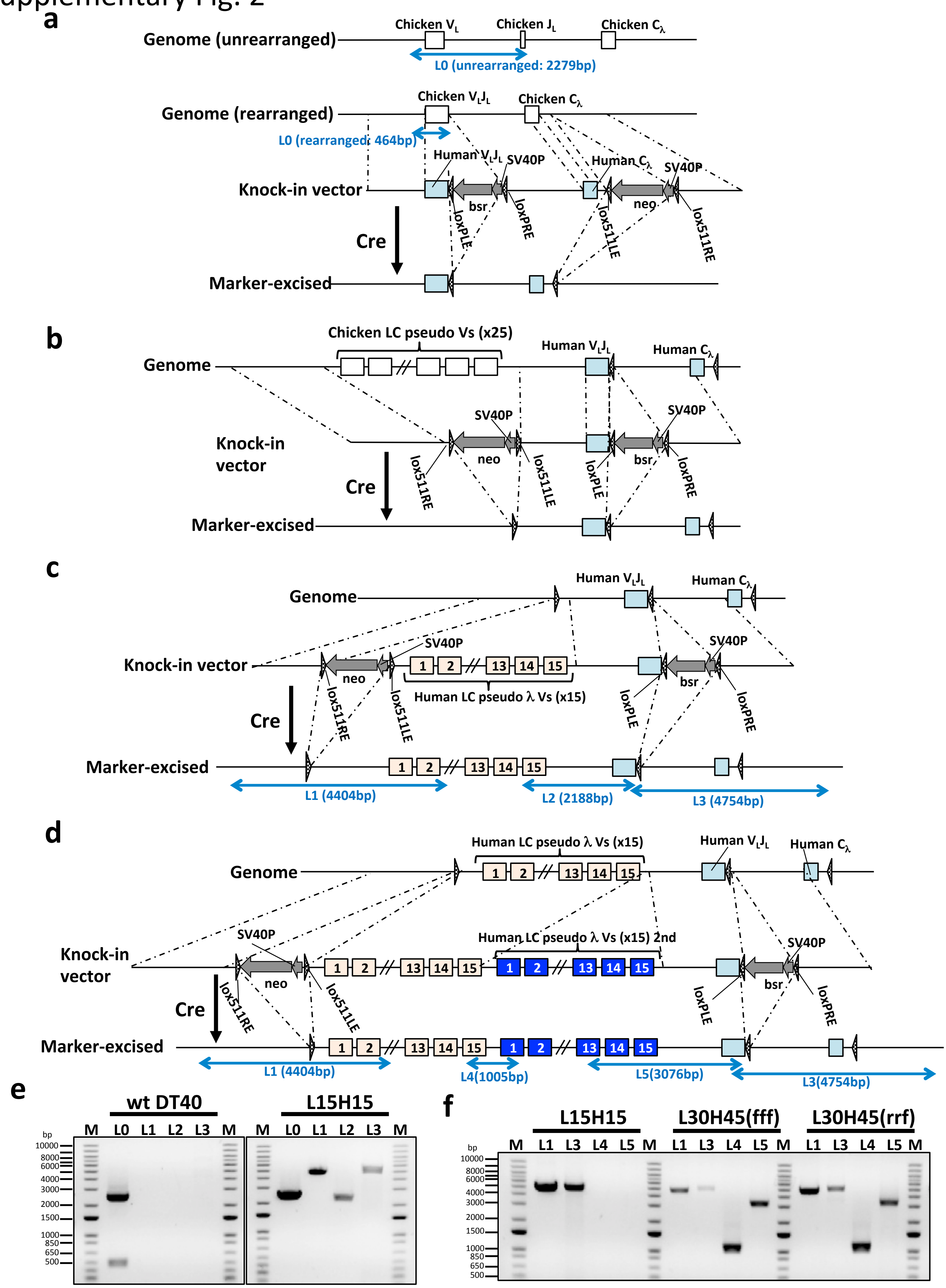

Supplementary Fig. 3

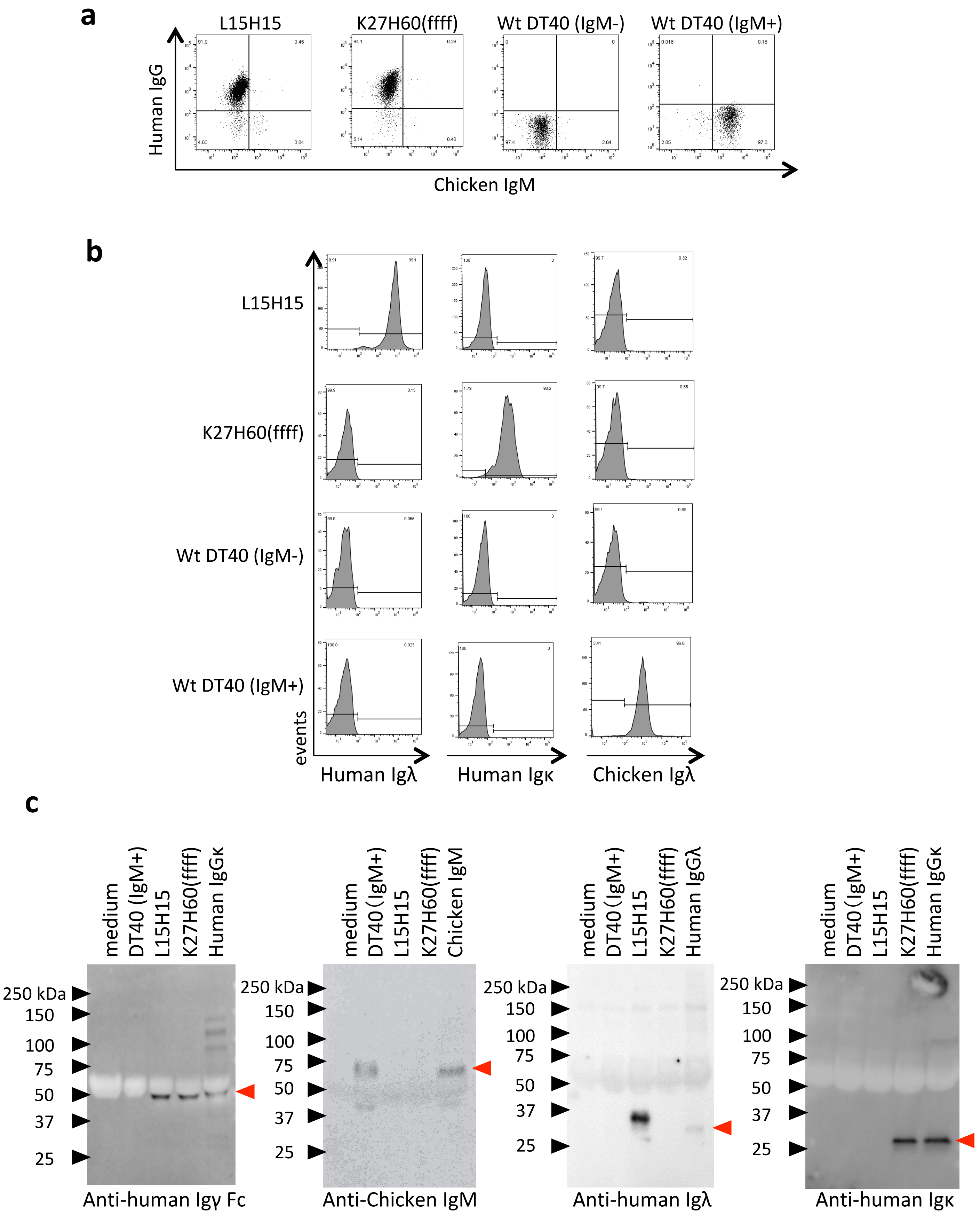

Supplementary Fig. 4

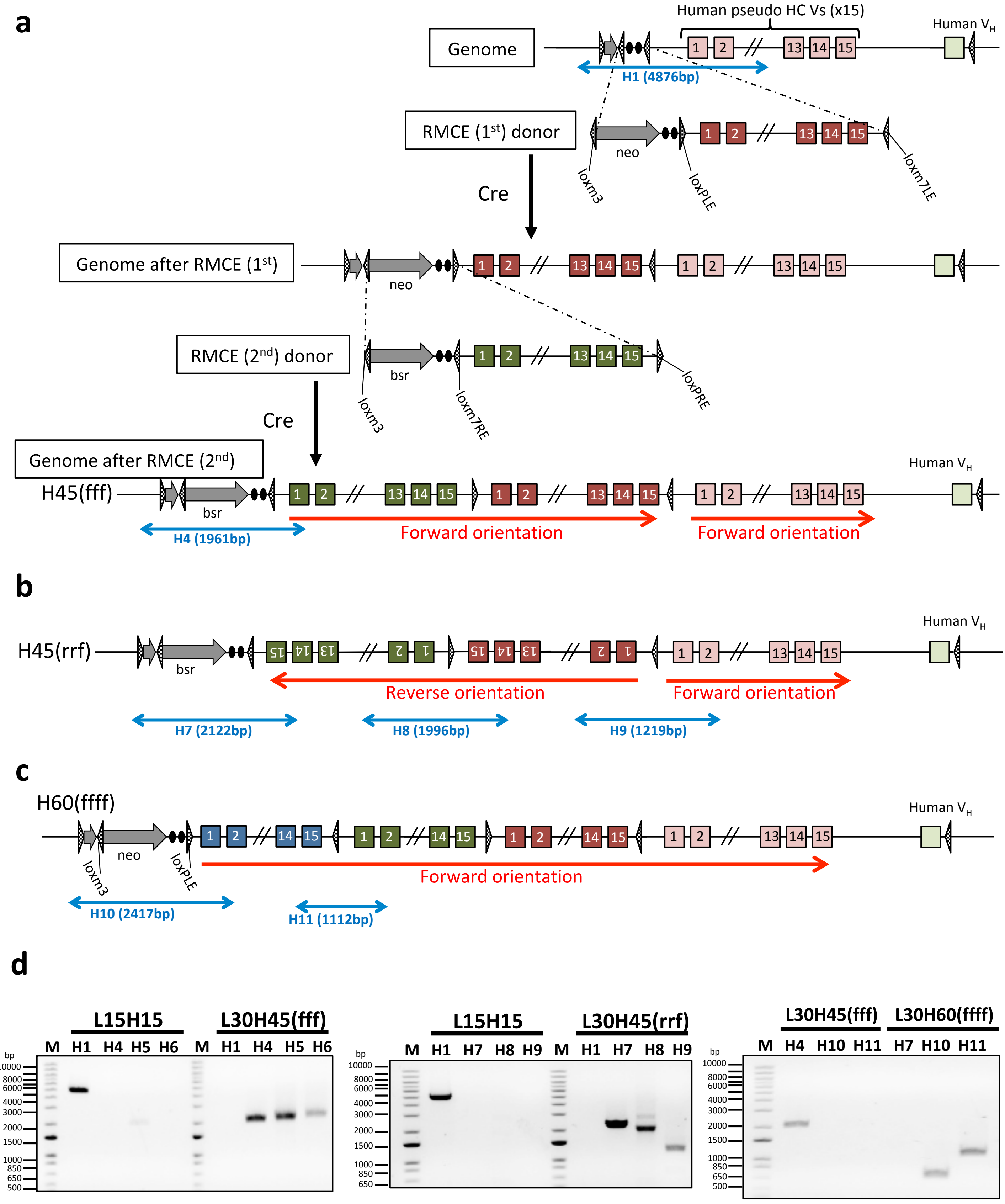

Supplementary Fig. 5

a

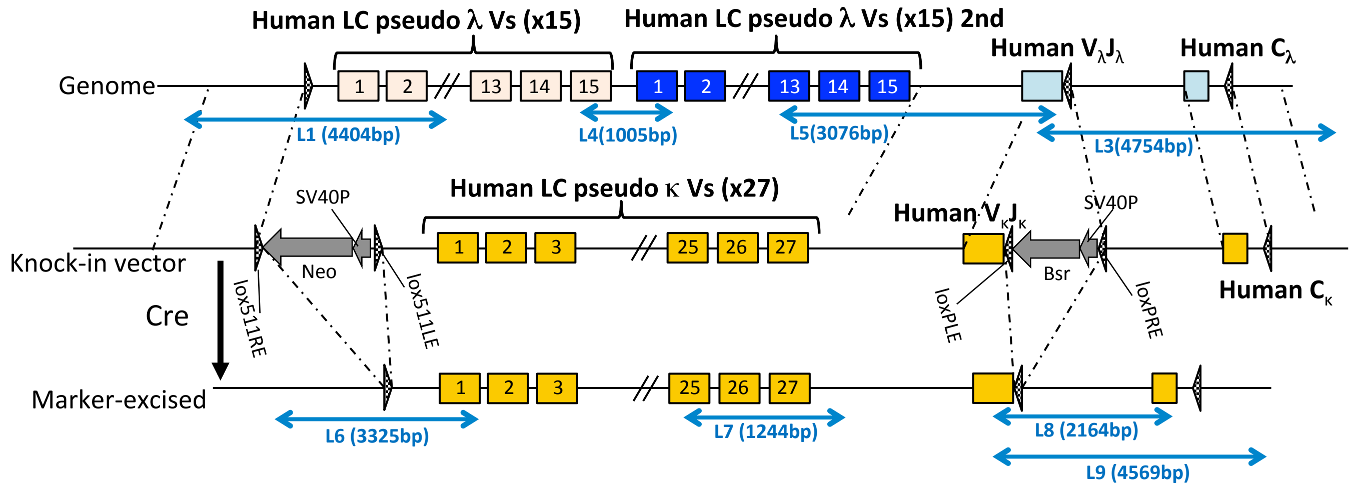

b

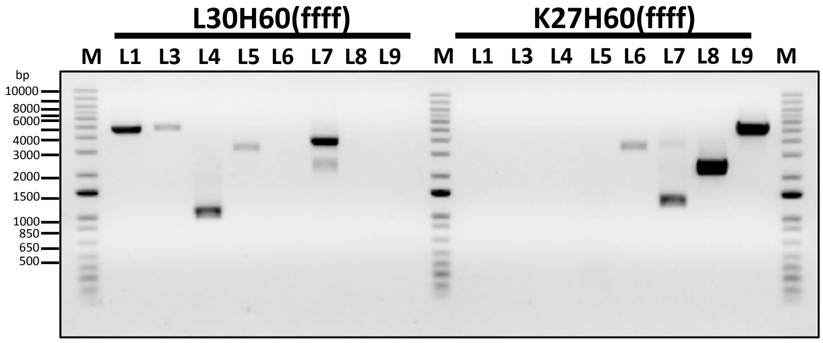

Supplementary Fig. 6

a

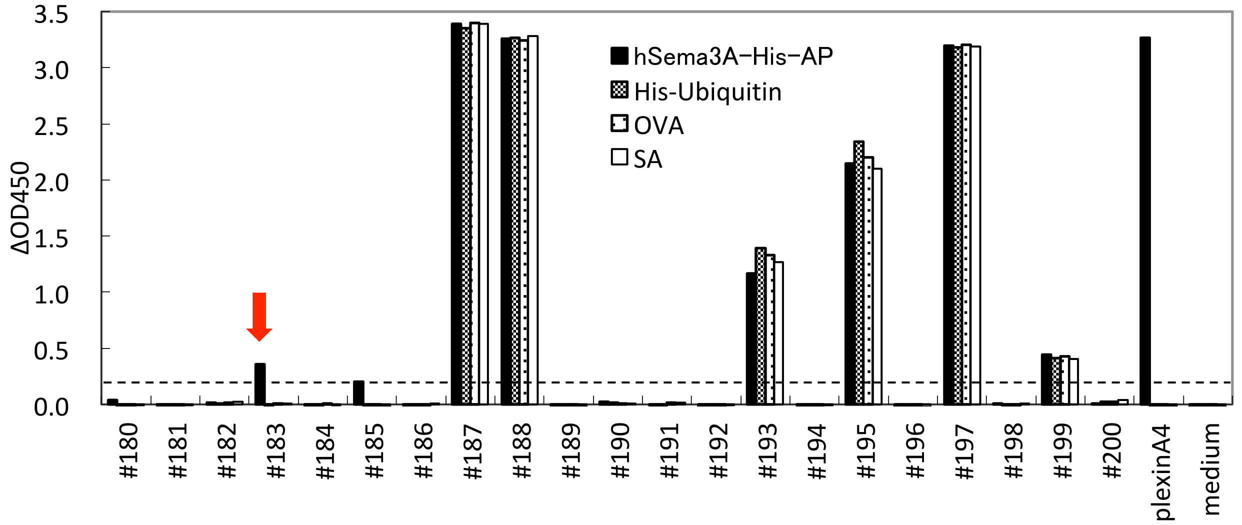

b

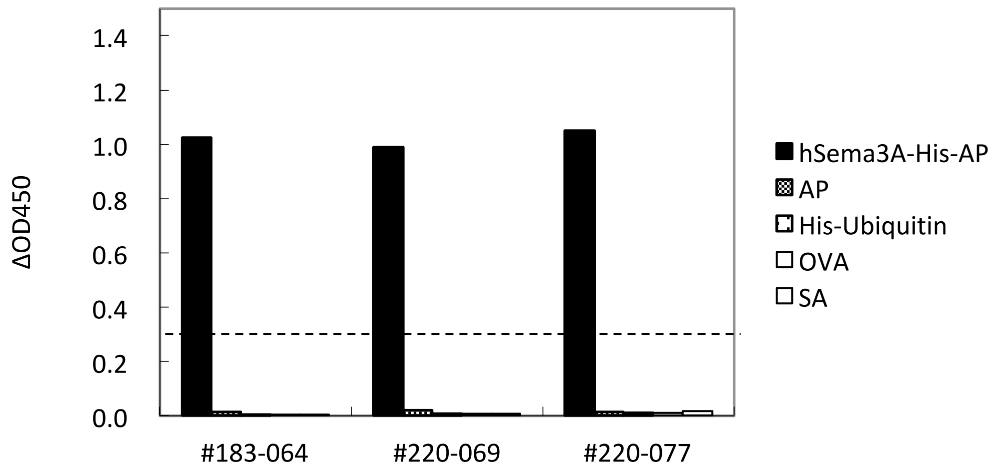

c

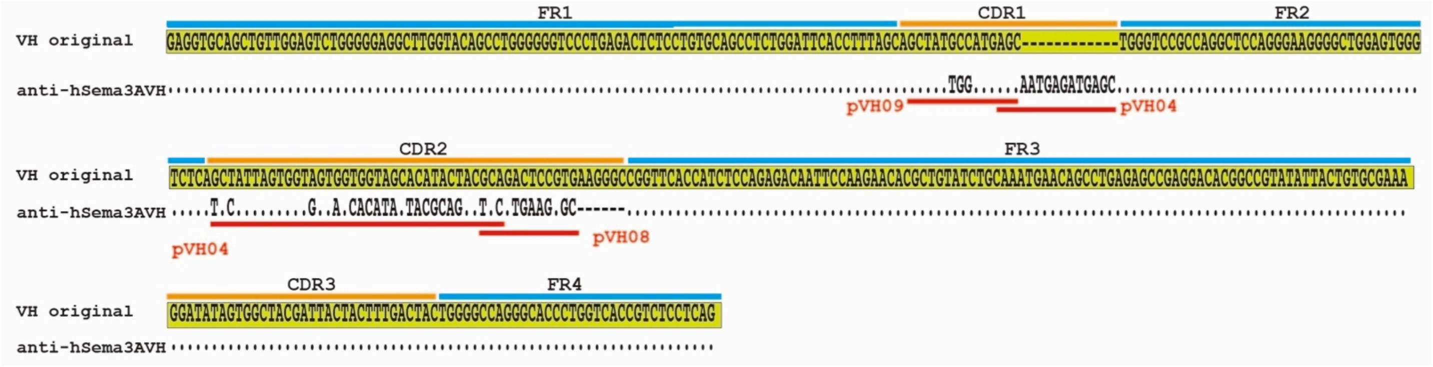

Supplementary Fig. 7

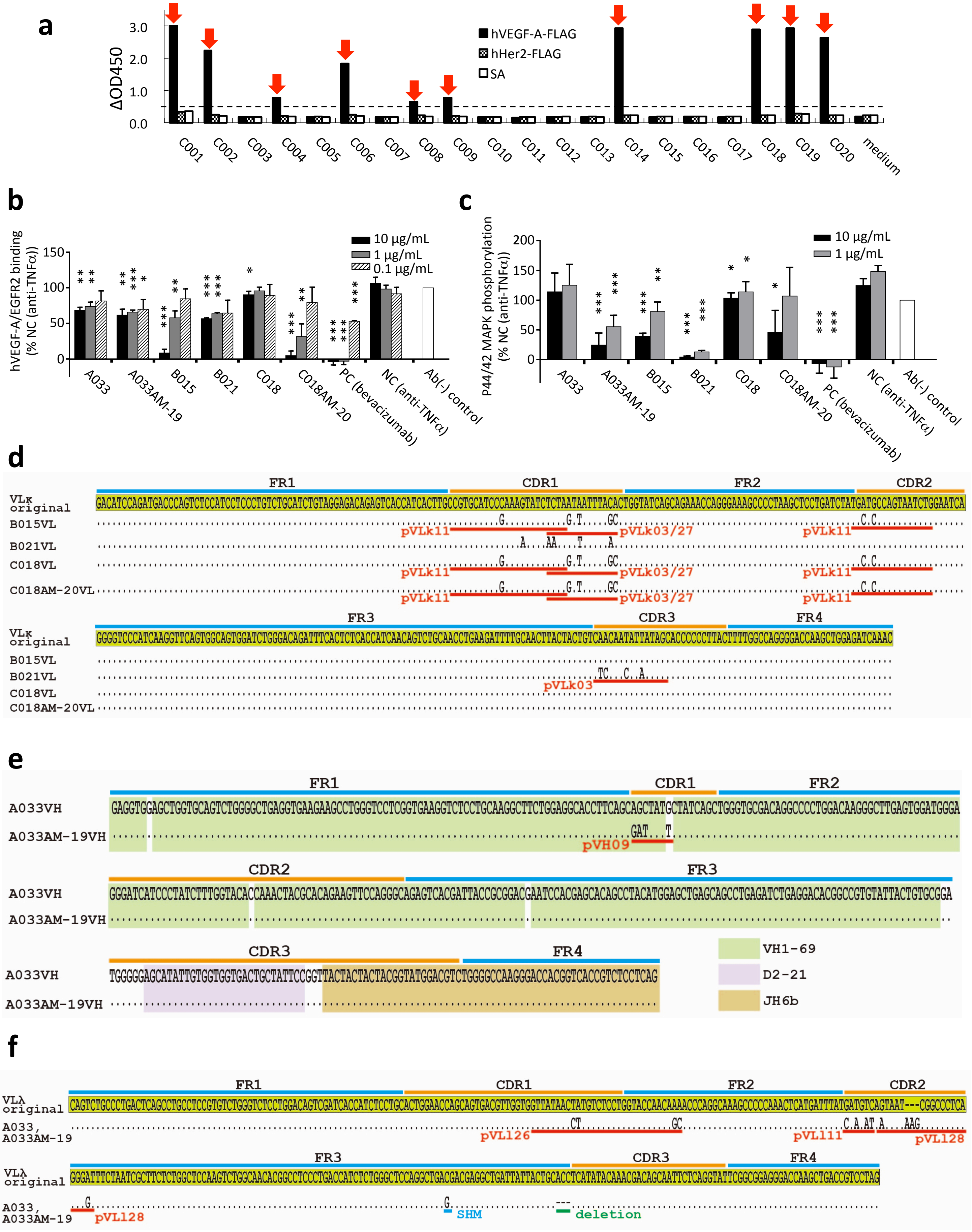

Supplementary Fig. 8

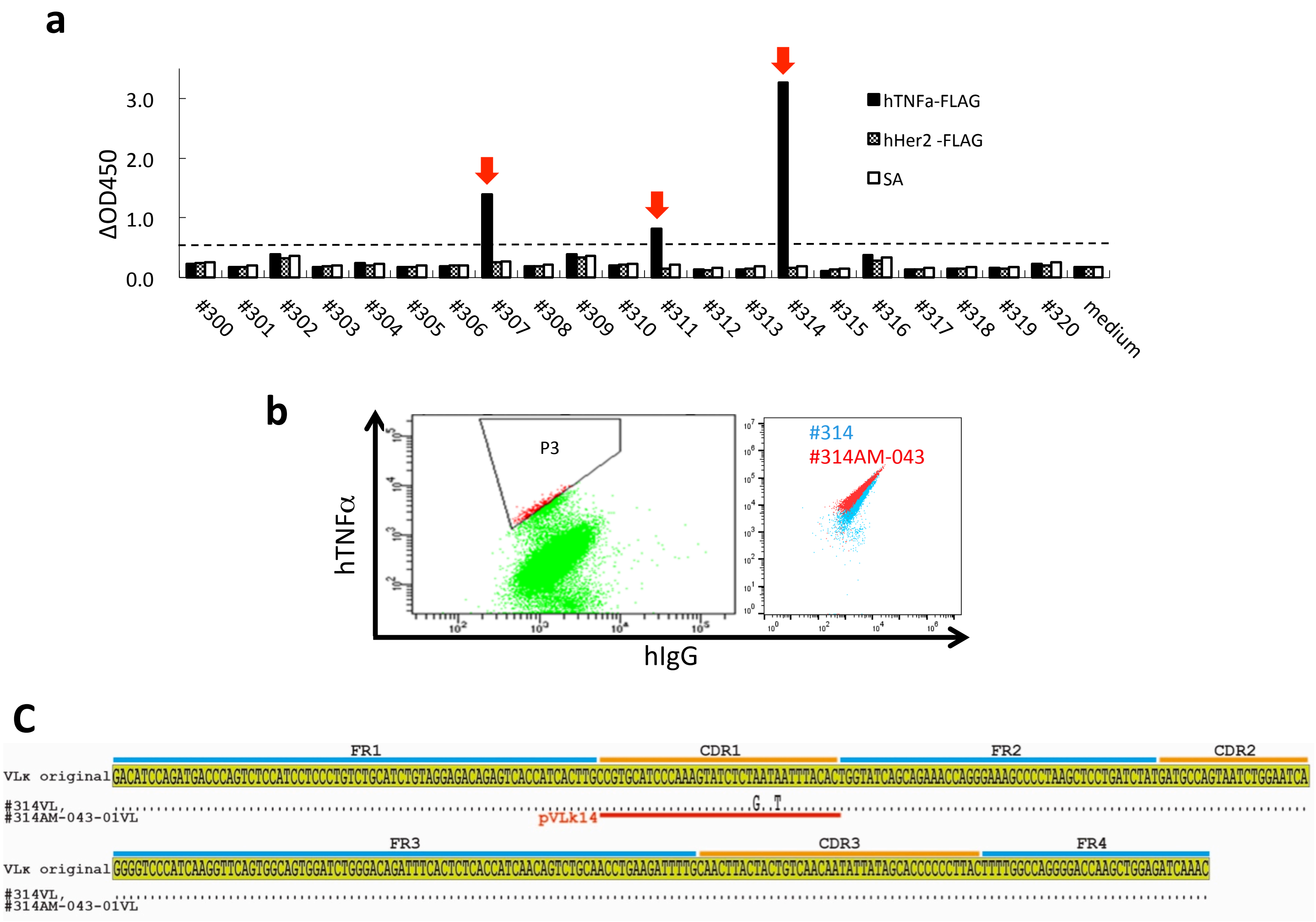

Supplement: Supplementary file 1 — Supplementary Fig.1, Supplementary Fig.2, Supplementary Fig.3, Supplementary Fig.4, Supplementary Fig. 5, Supplementary Fig.6, Supplementary Fig. 7 and Supplementary Fig.8 [file 41423_2020_440_MOESM1_ESM.pdf]
